# Impact of following saffron cultivation versus fallow management on yield components of five crop species in two semi-arid regions of Iran

**DOI:** 10.1186/s12870-026-09468-9

**Published:** 2026-07-17

**Authors:** Ali Abedinzadeh, Rahele Ghanbari Moheb Seraj, Ahmad Ghanbari, Mohammad Reza Asgharipour, Alireza Sirousmehr, Ahmad Ahmadian

**Affiliations:** 1 Department of Agronomy, Faculty of Agriculture, University of Zabol, Zabol, Iran; 2 Department of Horticultural Sciences, Faculty of Agriculture and Natural Resources, University of Mohaghegh Ardabili, Ardabil, Iran; 3 Department of Agronomy, Faculty of Agriculture, University of TorbatHeydarieh, Torbat Heydarieh, Iran

**Keywords:** Saffron (*Crocus sativus* L.), Crop rotation, Fallow, Field crops, Semi-arid regions, Yield components

## Abstract

Crop rotation and fallow management are widely used to maintain soil fertility and support sustainable land use. However, in semi-arid saffron-producing regions, there is limited evidence on whether post-saffron fields can be more productive and economically beneficial for subsequent crops than fallow lands. Addressing this gap is important for providing preliminary evidence to support crop rotation and field management decisions after saffron cultivation. This study evaluated the effects of post-saffron cultivation compared with fallow management on yield and yield-related traits of barley, cumin, chickpea, rapeseed, and sesame. The experiment was conducted during the 2022–2023 growing season in Torbat Heydarieh and Zaveh, two semi-arid regions of Iran. At each site, a split-plot arrangement based on a randomized complete block design with three replications was used, with field type as the main-plot factor and crop species as the subplot factor. Data were analyzed using combined analysis of variance across the two sites. Post-saffron cultivation increased seed yield in all five crops, with the magnitude of improvement ranging from 15.75% in cumin to 73.27% in rapeseed compared with fallow fields. Similar positive responses were observed for biological yield, with increases of 60.8% in barley, 18.71% in cumin, 46.51% in chickpea, 98.35% in rapeseed, and 23.87% in sesame. Harvest index showed a smaller and crop-specific response, increasing by 3.5% in barley and 7.13% in sesame, but decreasing by 2.46% in cumin and 1.73% in chickpea; in rapeseed, harvest index was not significantly affected by field type. The combined analysis showed that field type significantly affected most performance-related traits, while the city × field type and city × field type × crop interactions were not significant. Economic performance was also higher in post-saffron fields than in fallow fields, following the same general pattern observed for seed yield. Among the studied crops, cumin showed the highest economic performance, followed by sesame and rapeseed. Zaveh generally provided more favorable conditions than Torbat Heydarieh, particularly for seed yield, biological yield, and economic performance. Overall, under the conditions of this single-season field experiment conducted at two locations, the findings indicate that post-saffron fields may be used more productively than fallow lands by selecting suitable rotational crops, particularly cumin, sesame, and rapeseed. This strategy may contribute to improving land-use efficiency, farm income, and subsequent crop productivity in the studied regions. Moreover, saffron-based crop rotation may support integrated soil fertility management when combined with site-specific crop selection, balanced fertilization, and sustainable soil management practices. However, multi-year and multi-location validation is required before making broad practical recommendations for semi-arid saffron-producing regions.

## Introduction

Agricultural cultivation alters soil conditions, which can negatively affect plant performance in the long term. Continuous cultivation of crop monocultures can result in the build-up of pathogens in the soil, reducing yields of a broad range of crops from annuals to tree fruits. This condition is typically avoided by reducing the abundance of suitable plant hosts through fallow culture or crop rotation [1]. Fallow has been defined as a farming practice in which no crop is grown, and the growth of volunteer plants and weeds is controlled through cultivation or, in some systems, by chemical methods during a season when a crop might normally be grown [2]. Levels of soil organic matter, nutrients, and microbial biomass increase in cultivated fields that are left fallow for extended periods [3] and these successional changes in soil properties may influence growth and resistance to herbivores of crops cultivated following the fallow phase [4]. Fallow periods and crop rotation can influence soil fertility through several mechanisms, including the accumulation of plant residues, changes in root-derived carbon inputs, modification of nutrient cycling, and shifts in soil microbial communities. These processes may enhance soil organic matter formation, improve nutrient availability, and support microbial biomass, thereby contributing to soil fertility restoration and carbon storage before the establishment of subsequent crops [5, 6]. The composition of soil microbiomes is also known to shift over ecological succession [7], which is likely to affect the performance of the plants with which they interact.

Furthermore, crop rotation is the practice of growing different crop species successively on the same land across the growing seasons to maintain soil fertility and control weeds, diseases, and pests. A well-designed crop rotation can not only improve soil structure and organic matter but also reduce the pathogen and pest population in the field [8]. Therefore, crop rotation can serve as an effective management practice against soil-borne pathogens in perennial or long-term cropping systems and can play a crucial role in maintaining soil fertility [9]. Saffron is usually grown as a perennial crop and is affected by well-known problems associated with soil fertility decline, mainly due to weeds, biotic pathologies, and micronutrient deficiencies, which over the long term may result in substantial yield reductions and soil overexploitation [10]. Therefore, saffron cultivation should be included in crop rotation systems. Although information on the effects of previous crops on saffron yield and quality remains limited, 3 to 8 years are traditionally recommended before saffron is cultivated again on the same soil [10]. Continuous saffron cultivation on the same soil has also been associated with declining productivity and increased weed pressure, highlighting the importance of crop rotation for maintaining saffron performance. Therefore, in central Italy, saffron is profitably rotated with legumes and wheat. Saffron is well adapted to semi-arid regions [10] and may represent a profitable alternative crop in areas such as Iran.

Despite the agronomic importance of saffron in semi-arid regions, limited information is available on how fields previously cultivated with saffron affect the performance of subsequent crops compared with fallow fields. In particular, few studies have evaluated this effect across different crop species and contrasting local environmental conditions. Torbat Heydarieh and Zaveh were selected because they are important saffron-producing areas in northeastern Iran and represent semi-arid agricultural systems where decisions about fallow management, saffron-based rotation, and subsequent crop selection are highly relevant. The selected crops—rapeseed, barley, chickpea, cumin, and sesame—are commonly cultivated field crops in the region and differ in growth habit, resource demand, and economic value, making them suitable for evaluating crop-specific responses to post-saffron cultivation. Therefore, this study aimed to evaluate the effects of field type, including fallow management and post-saffron cultivation, on yield components, seed yield, biological yield, harvest index, and economic performance of five crop species—rapeseed (*Brassica napus* L.), barley (*Hordeum vulgare* L.), chickpea (*Cicer arietinum* L.), cumin (*Cuminum cyminum* L.), and sesame (*Sesamum indicum* L.)—in two semi-arid regions of Iran. The study also examined the effects of city, crop species, and their interactions to determine whether post-saffron cultivation improves subsequent crop performance compared with fallow management.

## Methods and materials

### Experimental materials

Seeds of rapeseed, barley, chickpea, cumin, and sesame were obtained from the Agricultural Jihad Organization of Khorasan Razavi province, Iran. The selected crop species included two improved cultivars, Okapi rapeseed and Goharan barley, and three locally adapted genotypes/landraces of chickpea, cumin, and sesame, which are commonly cultivated in the study regions. These crops were selected because of their agronomic importance, adaptation to semi-arid conditions, and potential suitability for crop rotation after saffron cultivation. The scientific name, cultivar or genotype, seed source, and main agronomic/yield-related characteristics of each crop species are presented in Table 1.

**Table 1:** Cultivation dates and seeding rates of the studied plant species

Plant species	Scientific name	variety	Cultivation date (Torbat Heydarieh)	Cultivation date (Zaveh)	Seeding rate (kgha^−1^)	seed density (seeds m⁻^2^)
Canola	*Brassica napus* L	Okapi	2022.10.02	2022.10.03	8	≈200
Barley	*Hordeum vulgare* L	Gohran	2022.10.26	2022.10.28	200	≈444
Chickpea	*Cicer arietinum* L	Local varieties	2023.03.06	2023.03.07	80	≈27
Cumin	*Cuminum cyminum* L	Local varieties	2023.03.06	2023.03.07	18	≈514
Sesame	*Sesamum indicum* L	Local varieties	2023.04.09	2023.04.10	6	≈200

### Study area and agroecological conditions

The experiment was conducted in two saffron-producing regions of Khorasan Razavi Province, northeastern Iran: Torbat Heydarieh (34.17°N, 59.12°E; 1450 m above sea level) and Zaveh, Sultan Suleiman village (35.18°N, 59.54°E; 1690 m above sea level). Both regions are located in semi-arid agroecological zones where saffron is traditionally cultivated and water availability is one of the major constraints for crop production. Based on 20-year long-term climatic averages, the minimum and maximum temperatures in Torbat Heydarieh are 8.2 and 37 °C, respectively, with a mean annual rainfall of 198 mm. In Zaveh, the corresponding minimum and maximum temperatures are 12.5 and 38.8 °C, respectively, and the mean annual rainfall is 190 mm. These climatic conditions indicate low precipitation and relatively high temperature during the growing season, making the regions suitable for evaluating crop rotation and fallow management under semi-arid conditions. In addition to the 20-year long-term climatic averages, climatic conditions during the experimental growing season were also considered. During the 2022–2023 growing season, both Torbat Heydarieh and Zaveh were characterized by semi-arid climatic conditions, with low precipitation, relatively large seasonal temperature fluctuations, and warm and dry conditions toward the end of the growing season. Monthly climatic data, including temperature, relative humidity, precipitation, and wind speed for both regions, are provided in Supplementary Table S1.

The soils of the study areas were generally loamy, slightly alkaline, and low in organic carbon and available nutrients. Torbat Heydarieh showed relatively more saline soil conditions, whereas Zaveh had more favorable non-saline soil conditions. These agroecological differences provided a suitable basis for comparing the performance of subsequent crops after saffron cultivation and fallow management across contrasting semi-arid environments.

### Experimental design and treatmentstructure

The field area was 300 m^2^ in both Torbat Heydarieh and Zaveh. In each region, 150 m^2^ was allocated to fallow land and 150 m^2^ to land previously cultivated with saffron. The saffron fields had been under saffron cultivation for eight years before the experiment. After the final saffron harvest, saffron corms were removed from the soil, and the experimental crops were sown one year after the termination of saffron cultivation. The fallow fields had remained uncultivated for five years before the experiment. During the first three years of the fallow period, the fields were left without crop production. In the last two years, shallow tillage was carried out to incorporate weed biomass into the soil and expose the soil surface to sunlight, thereby reducing weed growth and minimizing the uncontrolled effects of vegetation on soil fertility. No crop was grown in the fallow fields during this period. Thus, the two field types represented contrasting land-use histories: five-year fallow management and eight-year saffron cultivation followed by a one-year post-termination interval. In each region, 30 experimental plots, each 10 m^2^, were established based on two field types, five crop species, and three replications.

The experiment was arranged as a split-plot design based on a randomized complete block design at each site. Field type was assigned as the main-plot factor, with two levels: five-year fallow field and field following eight-year saffron cultivation. Crop species was assigned as the subplot factor, with five levels: barley, sesame, rapeseed, chickpea, and cumin. For the combined analysis, city/site was included as an additional factor to evaluate the effects of city, field type, crop species, and their interactions.

### Cultivation, fertilization and irrigation

In general, the cultivar used for each crop species, the sowing date in both the Torbat Heydarieh and Zaveh regions, and the seeding rates are presented in Table 1. The seeding rates (kg ha⁻^1^) were 8 for rapeseed, 200 for barley, 80 for chickpea, 18 for cumin, and 6 for sesame.

The fertilizers used in the experiment include urea (CO (NH2)2) containing 48% of the effective nitrogen source (NO3.NH4), triple superphosphate (CaH4 (PO4)2 with 21% P2O5, and potassium sulfate (K2SO4) containing 41% potassium. The fertilizer application rates for nitrogen, phosphorus, and potassium were 400, 300, and 300 g per plot, respectively, for all crop species. Phosphorus and potassium fertilizers were applied before planting.

In rapeseed plants, nitrogen fertilizer was applied at 200 g before planting, 100 g at the beginning of stem elongation, and 100 g at the beginning of flowering. In barley plants, nitrogen fertilizer was applied at 150 g before planting, 150 g at the tillering stage, and 100 g at the stem elongation stage. In cumin plants, nitrogen fertilizer was applied at 200 g at planting and 200 g at the vegetative growth stage. In sesame plants, nitrogen fertilizer was applied at 160 g before planting, 120 g at the stem elongation stage, and 120 g at the beginning of flowering.

All crop species were grown under irrigated conditions. Irrigation was managed according to the growth requirements of each crop species and the climatic conditions of Torbat Heydarieh and Zaveh. Because both regions have a semi-arid climate with mean annual precipitation of less than 200 mm, supplemental irrigation was applied at sensitive growth stages to prevent severe drought stress. Irrigation was performed using surface flooding with water from local wells. The irrigation schedule was adjusted based on soil moisture status, which was monitored using the gravimetric method, and the specific water requirement of each crop. Rapeseed and barley received three supplemental irrigations, each approximately 50 mm, at tillering or early vegetative growth, stem elongation, and grain-filling stages. Cumin and sesame received two supplemental irrigations, each approximately 40 mm, during vegetative growth and flowering. Chickpea received one supplemental irrigation of approximately 30 mm at the flowering stage because of its relatively higher drought tolerance. The irrigation strategy was applied to simulate local farming conditions while reducing excessive water use under semi-arid conditions.

### Plant growth and measurements

#### Soil analysis of the study regions

The physical and chemical properties of the soil were measured before and after the cultivation of each plant species. These properties included electrical conductivity (EC), soil texture, particle size distribution, soil pH, concentrations of nitrogen, phosphorus, potassium, calcium, magnesium and sodium, as well as some of soil organic compounds (Table 2).

**Table 2:** Analysis of soil composition before and after cultivation in both cities of Torbat-Heydarieh and Zaveh and both saffron and fallow fields

Torbat-Heydarieh city		Before culture		After culture-Saffron					After culture-Fallow				
**Analysis type**	**Optimal**	**Saffron**	**Fallow**	**Rapeseed**	**Barley**	**Chickpea**	**Cumin**	**Sesame**	**Rapeseed**	**Barley**	**Chickpea**	**Cumin**	**Sesame**
**EC**	Less than 2	3.22	5.19	3.65	3.03	3.65	4.01	4.16	5.10	5.03	4.65	5.01	4.86
**pH**	6–7.5	7.9	7.8	7.8	7.9	7.8	7.9	7.8	7.8	7.9	7.8	7.9	7.8
**T.N.V (%)**	Less than 10	21.25	22.25	22.25	22.75	22.25	22.25	23.75	22.25	22.45	22.35	22.25	23.15
**Clay (%)**	20	18	17	22	19	18	17	18	21	19	17	18	18
**Silt (%)**	40	48	56	50	51	53	53	50	58	55	52	53	56
**Sand (%)**	40	34	27	28	30	29	30	32	25	28	27	28	29
**Saturation (%)**	40	37.8	37.3	37.5	38.5	37.7	38	37.4	37.5	38.5	37.1	38	37.5
**Texture (%)**	Loam, Clay loam	Loam	Loam	Loam	Loam	Loam	Loam	Loam	Loam	Loam	Loam	Loam	Loam
**Organic carbon (%)**	More than 1.5	0.282	0.197	0.264	0.262	0.277	0.269	0.249	0.198	0.222	0.197	0.209	0.199
**Nitrogen (%)**	More than 0.15	0.025	0.016	0.023	0.023	0.024	0.023	0.025	0.014	0.015	0.017	0.016	0.014
**Phosphorus (ppm)**	15	16	10.5	13	14.5	14.5	15.5	15	9.5	10	8	9	10.5
**Potassium (ppm)**	300	321	227	319	320	317	321	319	225	227	224	222	225
**S.A.R**	Less than 13	7.1	10.13	7.6	8.6	8.03	9.8	10.4	10.3	9.6	8.03	10.8	11.4

**Zaveh city**		**Before culture**		**After culture-Saffron**					**After culture-Fallow**				
**Analysis type**	**Optimal**	**Saffron**	**Fallow**	**Rapeseed**	**Barley**	**Chickpea**	**Cumin**	**Sesame**	**Rapeseed**	**Barley**	**Chickpea**	**Cumin**	**Sesame**
**EC**	Less than 2	1.25	1.43	1.5	2.08	1.66	2.03	2.72	1.74	2.02	2.66	1.46	2.05
**pH**	6–7.5	7.8	7.9	7.9	7.9	7.8	7.8	7.9	7.7	7.8	7.9	7.9	7.9
**T.N.V (%)**	Less than 10	20.25	16	16	20	20.25	18.75	19	18.75	16.25	13.75	16.5	15.75
**Clay (%)**	20	16	8	12	13	13	13	13	10	12	10	9	8
**Silt (%)**	40	58	42	44	47	53	53	47	41	40	40	44	42
**Sand (%)**	40	29	50	44	40	34	34	40	49	48	50	47	50
**Saturation (%)**	40	37.4	38.3	37.9	38.5	38.3	38.3	38.4	38.1	38.1	38.5	37.8	38
**Texture (%)**	Loam, Clay loam	Loam	Loam	Loam	Loam	Loam	Loam	Loam	Loam	Loam	Loam	Loam	Loam
**Organic carbon (%)**	More than 1.5	0.269	0.182	0.267	0.254	0.271	0.244	0.248	0.184	0.201	0.183	0.191	0.185
**Nitrogen (%)**	More than 0.15	0.023	0.015	0.023	0.022	0.021	0.021	0.022	0.012	0.013	0.015	0.014	0.013
**Phosphorus (ppm)**	15	15.5	9	14	15	14.5	15.5	13	8	8	8.5	9	8.5
**Potassium (ppm)**	300	220	180	218	219	218.5	220	217.5	177	179	179.5	178	180
**S.A.R**	Less than 13	6.46	4.8	3.7	3.84	4.6	1.9	4.7	4.4	4.37	6.72	3.52	4.18

#### Analysis of yield and yield components in crop species

Yield and yield components were measured at physiological maturity. In each experimental plot, ten plants were randomly selected from the inner part of the plot, and the mean value of these plants was used as the representative value for that plot. The same general sampling protocol was applied to all crop species; however, crop-specific reproductive units, including pods in chickpea, umbels in cumin, clusters in barley, and pods/siliques in rapeseed and sesame, were recorded according to the morphology of each species. The number of seeds, pods, umbels, leaves and clusters was visually counted and recorded. Plant height of each species was measured using a meter with an accuracy of 1 mm.

To estimate seed yield and biological yield, a central harvest area of 1 m^2^ was harvested from each 10 m^2^ plot. This central harvest area was used to minimize border effects and provide a representative estimate of plot yield. For biological yield, the harvested aboveground biomass, including stems, leaves, and seeds, was dried at 70 °C for 48 h and then weighed. For seed yield, seeds were separated from the harvested plants, dried under the same conditions, and weighed. The biological yield and seed yield of each crop species were measured using a digital scale with an accuracy of 0.001g. The values obtained from the 1 m^2^ harvest area were converted to a hectare basis for yield reporting. Harvest index was calculated according to the following formula: Harvest index (%) = (Grain yield/Biological yield) × 100 [11].

To calculate the percentage changes of the studied traits, the minimum observed value for each trait was considered as the reference value. These indices were used as relative response indicators to compare the magnitude of trait responses across crop species and traits with different units and numerical scales. Seed yield variation, plant height variation, biological yield variation, and harvest index variation were calculated using the following equations:

$$\text{Seed yield variation} \left( \% \right)\ =\ [\left(\text{seed yield/minimum seed yield}\right)\ \times\ 100]\ -\ 100$$

$$\text{Plant height variation} \left( \%\right)\ =\ [\left(\text{plant height/minimum plant height}\right)\ \times\ 100]\ -\ 100$$

$$\text{Biological yield variation} \left( \%\right)\ =\ [\left(\text{biological yield/minimum biological yield}\right)\ \times\ 100]\ -\ 100$$

$$\text{Harvest index variation} \left( \% \right)\ =\ [\left(\text{harvest index/minimum harvest index}\right)\ \times\ 100]\ -\ 100$$

Economic performance was calculated based on the increase in seed yield relative to the minimum observed seed yield and the local market price of each crop. Before the economic calculation, all seed yield values were expressed in kg ha⁻^1^. Local crop prices were first recorded in Iranian Rial (IRR) during the 2022–2023 growing season and then converted to US dollars (USD) using the exchange rate of 490,957 IRR = 1 USD on August 18, 2024, obtained from the TGJU currency market database. Economic performance was expressed as million USD ha⁻^1^ and calculated as follows:

$$\text{Economic performance} \left( \text{million USD ha}^{-1}\right)\\\ =\ {[\text{seed yield} \left(\text{kg ha}^{-1}\right)-\ \text{minimum seed yield} \left(\text{kg ha}^{-1}\right)]\ \times\ \text{crop price} \left(\text{USD kg}^{-1}\right)}/1,000,000$$

For rapeseed and barley, because seed yield was expressed in tons, values were multiplied by 1000 to convert yield from tons to kilograms. The crop prices used for economic calculations were based on the average local market prices in Torbat Heydarieh and Zaveh during the 2022–2023 growing season.

### Statistical analyses

Statistical analyses of yield and yield components for the five crop species were conducted using R software version 3.5.3 (R Core Team, 2019) and RStudio version 1.1.463 (RStudio Team, 2018). The statistical analysis was performed at two levels. First, each crop species was analyzed separately. For each crop species, data from the two sites were analyzed together using a site-combined ANOVA based on a split-plot arrangement in a randomized complete block design with three replications. In these crop-wise analyses, city/site, field type, and the city × field type interaction were included in the model. Treatments were considered as fixed effects, whereas replications/blocks were considered as random effects. City/site was tested using the first error term (Ea), while field type and the city × field type interaction were tested using the second error term (Eb). Separate ANOVA tables and mean-comparison figures were prepared for each crop species.

Second, an overall combined ANOVA across all five crop species was performed to compare performance-related traits among crop species, city/site, field type, and their relevant interactions. The results of this overall combined analysis were presented separately in the combined ANOVA table and corresponding figures. Mean comparisons were performed using Duncan’s multiple range test implemented in the agricolae package [12] after significant ANOVA results at a significance level of 5% (P ≤ 0.05). Significance levels were consistently reported as *P* < 0.05, *P* < 0.01, and ns, not significant. Exact P-values were also calculated where applicable and provided as a supplementary file.

In addition, Pearson correlation analysis was performed to evaluate the relationships between post-cultivation soil properties and crop morpho-physiological and performance-related traits. For this analysis, soil and crop data were matched at the city/site × field type × crop species level, and mean values across replications were used. Pearson correlation coefficients (r) and their corresponding P-values were calculated using the cor.test function in the base stats package of R. Correlation matrices and heatmaps were prepared using the ggplot2 package. Correlations were considered significant at *P* < 0.05 and highly significant at *P* < 0.01. All figures and tables were prepared using Microsoft Word and Excel 2013.

## Results

### Analysis of soil composition

Before cultivation, soil analysis was performed in both post-saffron and fallow fields in Torbat Heydarieh and Zaveh to characterize the initial physical and chemical soil conditions. After cultivation, soil properties were also measured separately for each crop species in both field types and cities.

In Torbat Heydarieh, the initial soil conditions differed clearly between the post-saffron and fallow fields. The post-saffron field had lower EC than the fallow field, indicating relatively lower salinity. It also had higher organic carbon, nitrogen, phosphorus, and potassium contents, while SAR was lower than in the fallow field. In contrast, the fallow field showed higher salinity, lower nutrient status, and higher SAR. Both field types had slightly alkaline pH, high lime content, loamy texture, and low organic carbon compared with the optimal range. After cultivation, only minor changes were observed in most soil properties in Torbat Heydarieh. However, post-saffron plots generally maintained higher organic carbon and NPK contents and lower EC than fallow plots, suggesting a relatively better soil fertility status in the fields previously cultivated with saffron.

In Zaveh, both post-saffron and fallow fields had lower EC values than those observed in Torbat Heydarieh, indicating more favorable non-saline soil conditions. Similar to Torbat Heydarieh, the initial post-saffron soil in Zaveh contained higher organic carbon, nitrogen, phosphorus, and potassium than the fallow soil, although all values remained below the optimal range for organic carbon and nitrogen. After cultivation, the post-saffron plots generally preserved higher nutrient contents than the fallow plots across the five crop species. Among the post-saffron plots, chickpea showed the highest organic carbon value, while cumin maintained relatively higher phosphorus and potassium contents. In the fallow plots, only slight increases in phosphorus and potassium were observed after cultivation, whereas organic carbon and nitrogen remained low.

Overall, the soil results indicate that the post-saffron fields had a more favorable fertility status than the fallow fields in both cities, particularly in terms of organic carbon and available N, P, and K. The difference was more evident in Torbat Heydarieh, where fallow soil also showed higher salinity. These soil conditions may partly explain the better yield performance of the subsequent crops grown in post-saffron fields compared with fallow fields.

### ANOVA analysis based on a split plot design

In this experiment, the effects of field type (saffron and fallow) and city (Torbat Heydarieh and Zaveh) on the morphological and physiological traits of five types of crop species (barley, cumin, chickpea, rapeseed, sesame) were investigated.

#### Morphological and physiological characteristics of barley

The results of the analysis of variance showed that there were no significant differences among the measured traits of barley with respect to city. In contrast, under the field type treatment, significant differences were observed for most traits, except leaves number and clusters number per plant. Among the significant traits, harvest index and harvest index variation were significant at the 0.05 level, whereas the remaining traits were significant at the 0.01 level. Moreover, the interaction between field type and city was not significant in any of the measured traits (Table 3).

**Table 3:** Analysis of variance (ANOVA) for morphological and physiological traits of barley under different field types and cities

Sources of Variation	df	Plant height	Number of leaves per plant	Number of clusters per plant	Number of seeds per cluster	Number of seeds per plant	Seed yield	Biological yield	Harvest index	Yield variation	Economic performance	Height variation	Biologicalyield variation	Harvest index variation
block	2	23.25	0.333	0.250	15.75	533.6	0.120	0.685	0.174	345.5	20,417	48.83	332.2	1.119
Ea	2	4.75	0.333	0.083	1.750	86.1	0.034	0.273	0.319	99.6	58,854	9.98	132.6	2.046
Field	1	1408^**^	4.083^ns^	3.000^ns^	243.0^**^	16,428^**^	4.750^**^	33.73^**^	5.796^*^	13,584^**^	80,279^**^	2958^**^	16,366^**^	37.13^*^
Eb	4	14.67	0.666	0.500	1.917	671.2	0.185	1.230	0.321	531.1	31,389	30.81	596.5	2.057

^*^significance at *P* < 0.05, ^**^significance at *P* < 0.01, ^ns^ no significance

#### Morphological and physiological characteristics of cumin

In cumin, significant differences were observed among city types for number of umbels per plant, number of seeds per plant, seed yield, biological yield, harvest index, yield variations, economic performance, biological yield variations and harvest index variations at the 0.05 level, while other traits showed no significant differences. For field type, highly significant difference (*P* < 0.01) was observed in plant height, biological yield, harvest index, height variations, biological yield variations and harvest index and significant differences (*P* < 0.05) in number of umbels per plant, number of seeds per umbel, number of seeds per plant, seed yield, yield variations and economic performance. The interaction between field type and city type was significant for plant height and height variations at the 0.01 level, and for harvest index and harvest index variations at the 0.05 level no other traits were significantly influenced (Table 4).

**Table 4:** Analysis of variance (ANOVA) for morphological and physiological traits of cumin under different field types and cities

Sources of Variation	df	Plant height	Number of umbel per plant	Number of seeds per umbel	Number of seeds per plant	Seed yield	Biological yield	Harvest index	Yield variations	Economic performance	Height variations	Biological yield variations	Harvest index variations
Block	2	13.08	4.083	25.08	39,315	110,440	404,713	0.055	771.5	4329.3	452.7	791.6	0.4211
City	1	52.08^ns^	6.750^*^	300.0^ns^	151,425^*^	239,984^*^	1,421,408^*^	2.201^*^	3353.1^*^	18,815^*^	1802.1^ns^	2780.4^*^	16.72^*^
Ea	2	3.583	0.250	20.25	4901	7912	64,736	0.024	110.5	620.27	123.99	126.63	0.188
Field	1	52.08^**^	2.083^*^	147.0^*^	59,361^*^	88,924^*^	880,208^**^	2.594^**^	1242.4^*^	6971.6^*^	1802.1^**^	1721.8^**^	19.71^**^
City × Field	1	6.75^**^	0.750^ns^	3.000^ns^	3267^ns^	6211^ns^	59080^ns^	0.108^*^	86.8^ns^	486.92^ns^	233.5^**^	115.5^ns^	0.822^*^
Eb	4	0.167	0.166	16.50	3460	4704	31,699	0.011	262.9	368.77	5.77	62.01	0.084

^*^significance at *P* < 0.05, ^**^significance at *P* < 0.01, ^ns^ no significance

#### Morphological and physiological characteristics of chickpea

According to the ANOVA results in chickpeas, no significant differences were observed among treatments for city type or for the city × field interaction in any of the studied morpho-physiological traits. For the field type treatment, the majority of traits exhibited highly significant differences at the 0.01 probability level, plant height and height variations were significant at the 0.05 level, and only number of seeds per pod showed no significant difference (Table 5).

**Table 5:** Analysis of variance (ANOVA) for morphological and physiological traits of chickpea under different field types and cities

Sources of Variation	df	Plant height	Number of pods per plant	Number of seeds per pod	Number of seeds per plant	Seed yield	Biological yield	Harvest index	Yield variations	Economic performance	Height variations	Biological yield variations	Harvest index variations
Block	2	30.00	12.00	1.9721e-31	12.00	1388	5202	0.02257	88.9	2810	360.68	80.1	0.0983
City	1	0.001^ns^	3.00^ns^	1.9721e-31^ns^	3.00^ns^	1541^ns^	7450^ns^	0.04688^ns^	98.8^ns^	3121.2^ns^	0.00^ns^	114.7^ns^	0.2040^ns^
Ea	2	21.00	4.00	1.9721e-31	4.00	301	1214	0.00527	19.3	609.69	249.70	18.7	0.0230
Field	1	243.0^*^	385.3^**^	1.9721e-31^ns^	385.3^**^	106,408^**^	494,914^**^	2.17601^**^	6820.0^**^	215,480^**^	2889.4^*^	7618.3^**^	9.4721^**^
Eb	4	11.667	0.67	1.9721e-31	0.67	115	667	0.03856	7.4	233.04	138.72	10.3	0.1678

^*^significance at *P* < 0.05, ^**^significance at *P* < 0.01, ^ns^ no significance

#### Morphological and physiological characteristics of rapeseed

The analysis of variance for rapeseed indicated that city and the interaction between field type and city had no significant effects on any of the evaluated morpho-physiological traits. In contrast, field type had a significant effect on most traits; however, harvest index and harvest index variations showed no significant differences. Among the significant traits, the number of seeds per pod was significant at the 0.05 probability, whereas all remaining traits exhibited highly significant differences at the 0.01 level. (Table 6).

**Table 6:** Analysis of variance (ANOVA) for morphological and physiological traits of rapeseed under different field types and cities

Sources of Variation	df	Plant height	Number of pods per plant	Number of seeds per pod	Number of seeds per plant	Seed yield	Biological yield	Harvest index	Yield variations	Economic performance	Height variations	Biological yield variations	Harvest index variations
Block	2	18.25	894.2	3.583	181,927	0.00842	0.0049	6.824	212.1	10.31	38.33	16	79.86
City	1	3.00^ns^	114.1^ns^	0.083^ns^	31314^ns^	0.01153^ns^	0.0034^ns^	7.395^ns^	290.6^ns^	14.127^ns^	6.30^ns^	11^ns^	86.55^ns^
Ea	2	4.75	363.6	2.583	488,888	0.01640	0.0068	18.765	413.3	20.094	9.98	22	219.63
Field	1	616.3^**^	29,304.1^**^	90.750^*^	52,697,634^**^	0.90970^**^	9.5819^**^	77.318^ns^	22,920.2^**^	1114.4^**^	1294.5^**^	31,831^**^	904.94^ns^
Eb	4	12.17	252.6	5.750	579,242	0.01188	0.0068	20.818	299.4	14.557	25.55	23	243.66

^*^significance at *P* < 0.05, ^**^significance at *P* < 0.01, ^ns^ no significance

#### Morphological and physiological characteristics of sesame

The analysis of variance for sesame showed that city type had a significant effect on number of pods per plant, number of seeds per plant, seed yield, biological yield, yield variations, economic performance and biological yield variations at the 0.01 probability level, while number of seeds per pod was significant at the 0.05 level, no significant differences were observed for the remaining traits. All measured traits were significantly influenced by field type; number of seeds per pod and number of seeds per plant were significant at *P* < 0.01, whereas the other traits were significant at *P* < 0.05. However, the interaction between field type and city was not significant for any trait (Table 7).

**Table 7:** Analysis of variance (ANOVA) for morphological and physiological traits of sesame under different field types and cities

Sources of Variation	df	Plant height	Number of pods per plant	Number of seeds per pod	Number of seeds per plant	Seed yield	Biological yield	Harvest index	Yield variations	Economic performance	Height variations	Biological yield variations	Harvest index variations
Block	2	52.58	20.333	14.58	209,528	22,954	710,091	0.1119	349.9	165.85	78.20	395.92	3.26
City	1	300.0^ns^	126.7^**^	168.75^*^	1,989,416^**^	210,145^**^	1,527,960^**^	11.48^ns^	3202.9^**^	1518.3^**^	446.16^ns^	851.93^**^	334.87^ns^
Ea	2	42.25	1.00	1.75	5469	940	14,540	0.814	14.3	6.7939	62.83	23.73	0.188
Field	1	1452.0^*^	216.7^*^	352.08^**^	3,645,416^**^	347,480^*^	4,775,408^*^	5.713^*^	5296.1^*^	2510.5^*^	2159.4^*^	166.57^*^	19.71^*^
Eb	4	93.42	13.667	13.83	164,437	17,473	361,241	0.495	266.3	126.24	138.93	14.44	0.084

^*^significance at *P* < 0.05, ^**^significance at *P* < 0.01, ^ns^ no significance

#### The effect of field type on morphological and physiological traits of barley

**Fig. 1:**
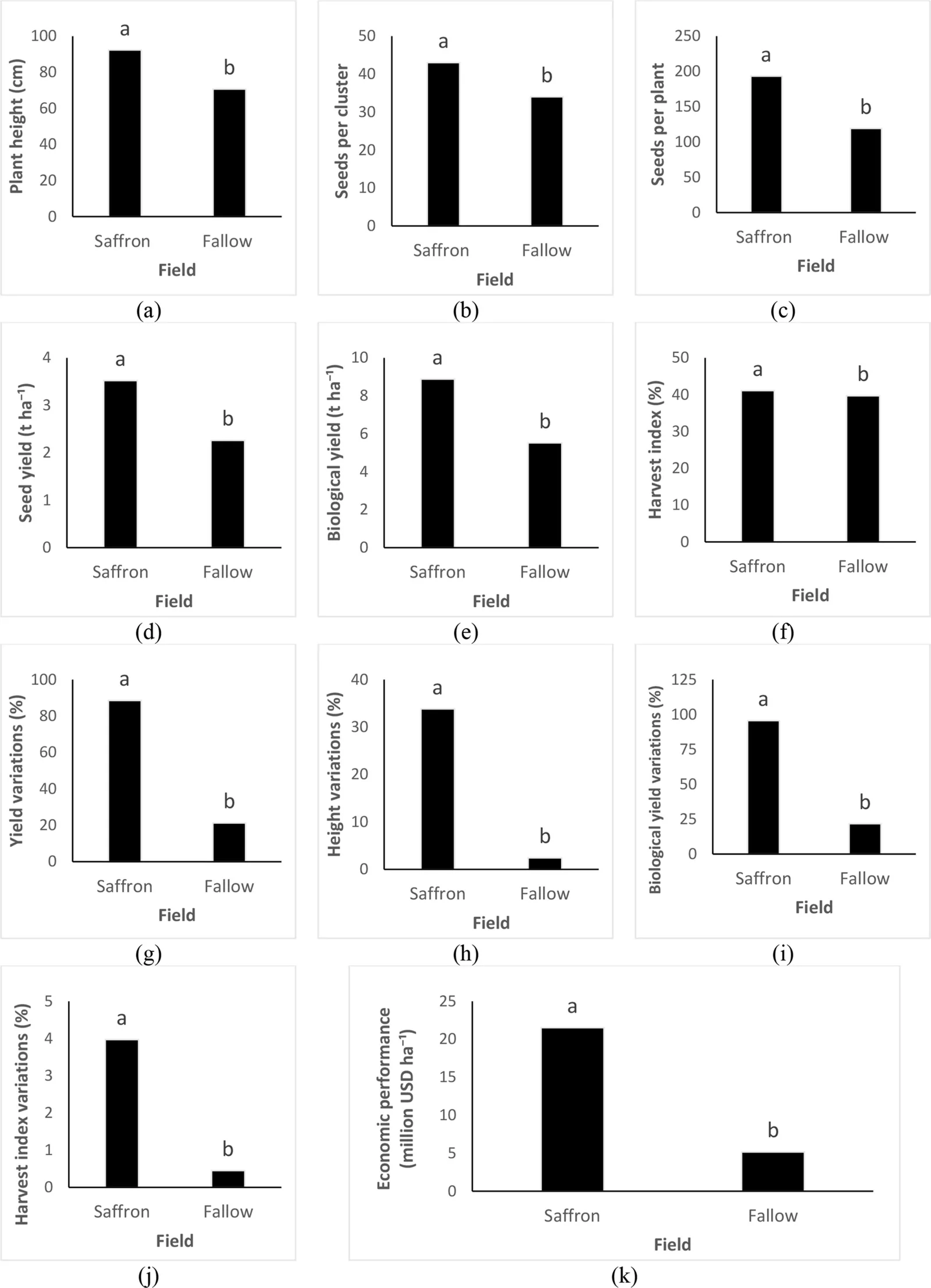
Simple and interaction effects of field and city type on morpho-physiological traits of barley

Mean comparisons (Fig. 1) showed that traits with significant differences had higher values in barley grown in the saffron field than in the fallow field. Thus, compared with the fallow field, the saffron field resulted in percentage increases of 23.4% in plant height, 26.4% in number of seeds per cluster, 62.1% in number of seeds per plant, 55.7% in seed yield, 60.8% in biological yield, 3.5% in harvest index, as well as 317.2%, 1302.9%, 339.8%, and 782.2% increases in yield, height, biological yield, and harvest index variations, respectively, and a 317.4% increase in economic performance.

#### The effect of field type, city type and field ×city interaction on morphological and physiological characteristics of cumin

**Fig. 2:**
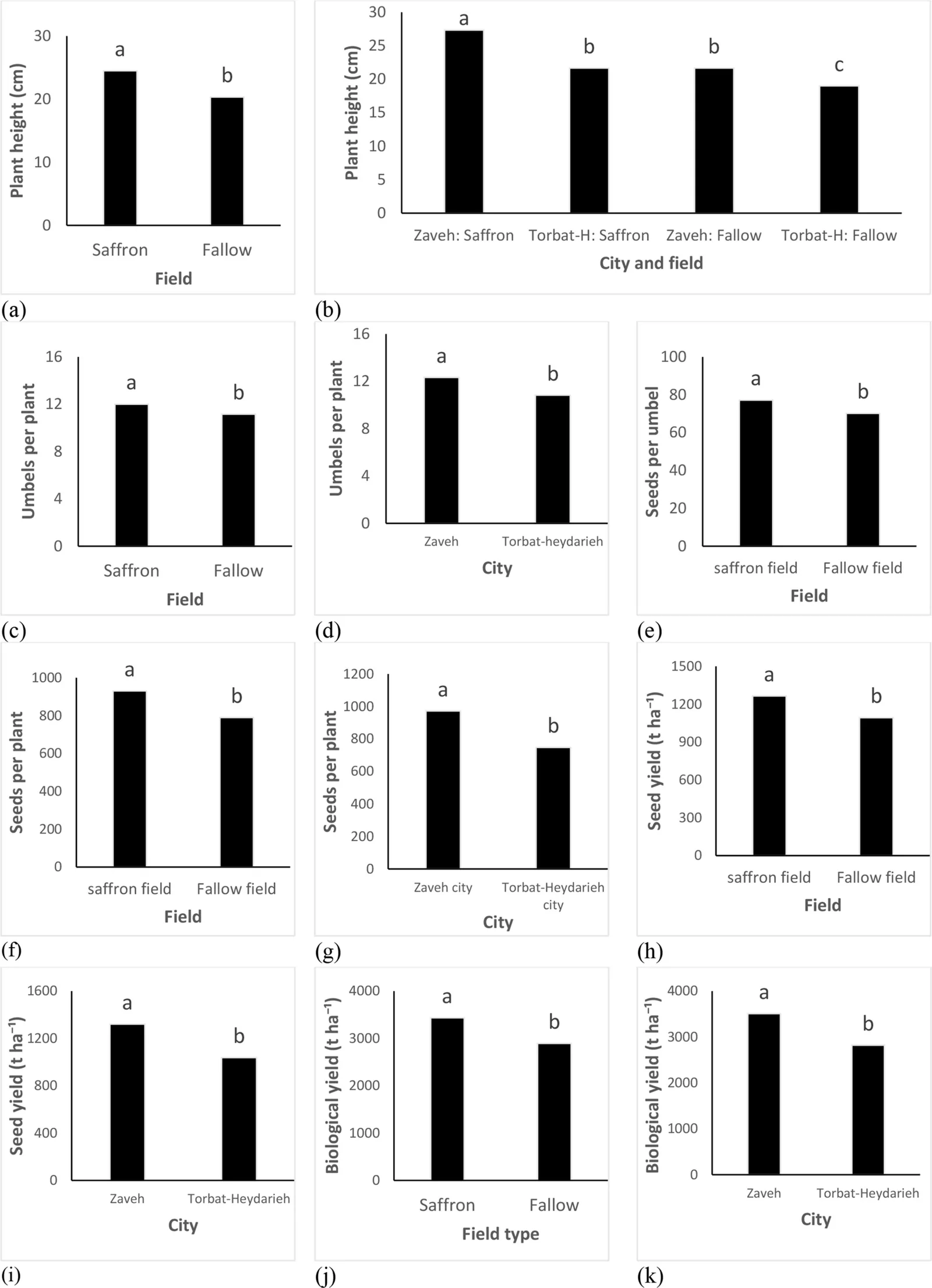

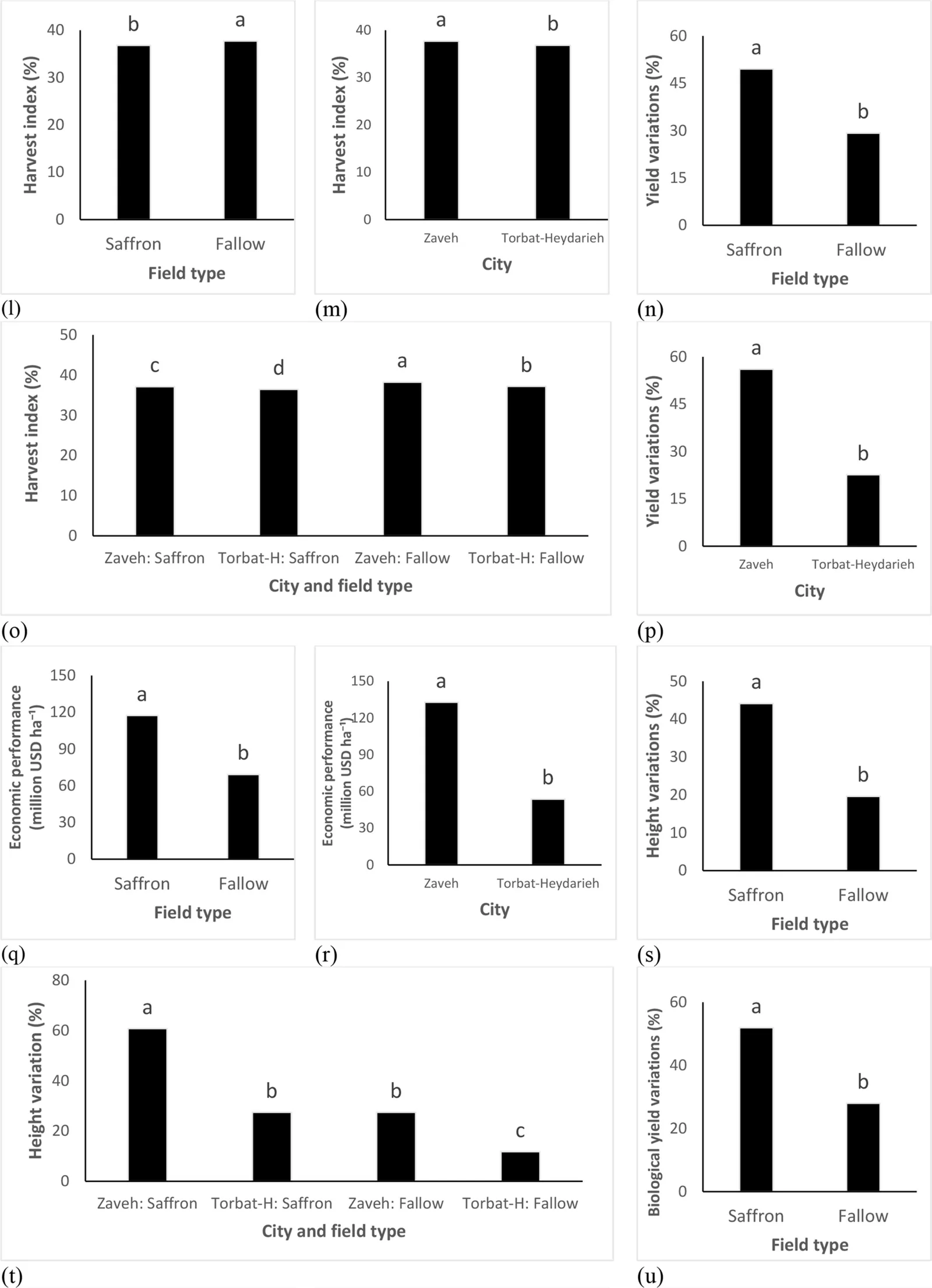

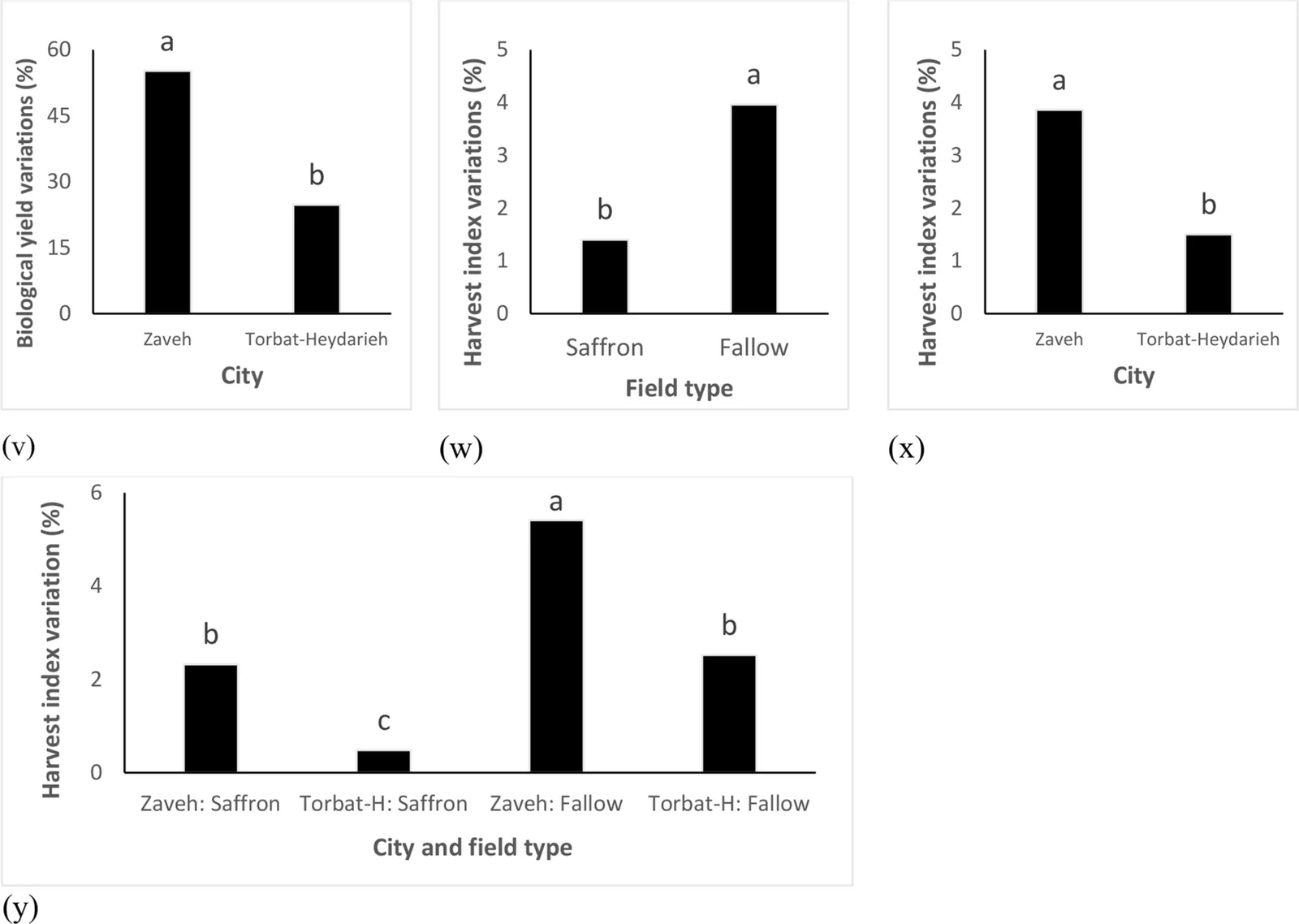
Simple and interaction effects of field type and cultivation city on morpho-physiological traits of cumin

The mean comparison results for cumin indicated that, under the field type treatment, the saffron field outperformed the fallow field for all evaluated traits, except harvest index and harvest index variation. The percentage changes of traits in the saffron field relative to the fallow field were as follows: plant height (20.51%), number of umbels per plant (7.50%), number of seeds per umbel (9.97%), number of seeds per plant (17.81%), seed yield (15.75%), biological yield (18.71%), harvest index (− 2.46%), yield variation (69.71%), economic performance (69.69%), height variation (125.05%), biological yield variation (85.59%), and harvest index variation (− 64.60%) (Fig. 2).

The mean comparison results showed that all significant traits had higher values in Zaveh than in Torbat Heydarieh. Compared with Torbat Heydarieh, Zaveh exhibited increases of 13.85% in the number of umbels per plant, 30.04% in the number of seeds per plant, 27.25% in seed yield, 24.40% in biological yield, 2.33% in harvest index, 147.5% in yield variation, 147.5% in economic performance, 123.1% in biological yield variation, and 157.3% in harvest index variation.

The mean comparison of the city × field interaction showed that plant height and height variation were highest in the Zaveh × saffron field combination and lowest in Torbat Heydarieh × fallow field, with percentage differences of 43.84% and 416.8%, respectively. In contrast, harvest index and harvest index variation reached their highest values in Zaveh × fallow field and the lowest values in Torbat Heydarieh × saffron field, with corresponding differences of 4.91% and 1027.08% (Fig. 2).

#### The effect of field type, city type and their interaction (field × city) on morphological and physiological characteristics of chickpea

**Fig. 3:**
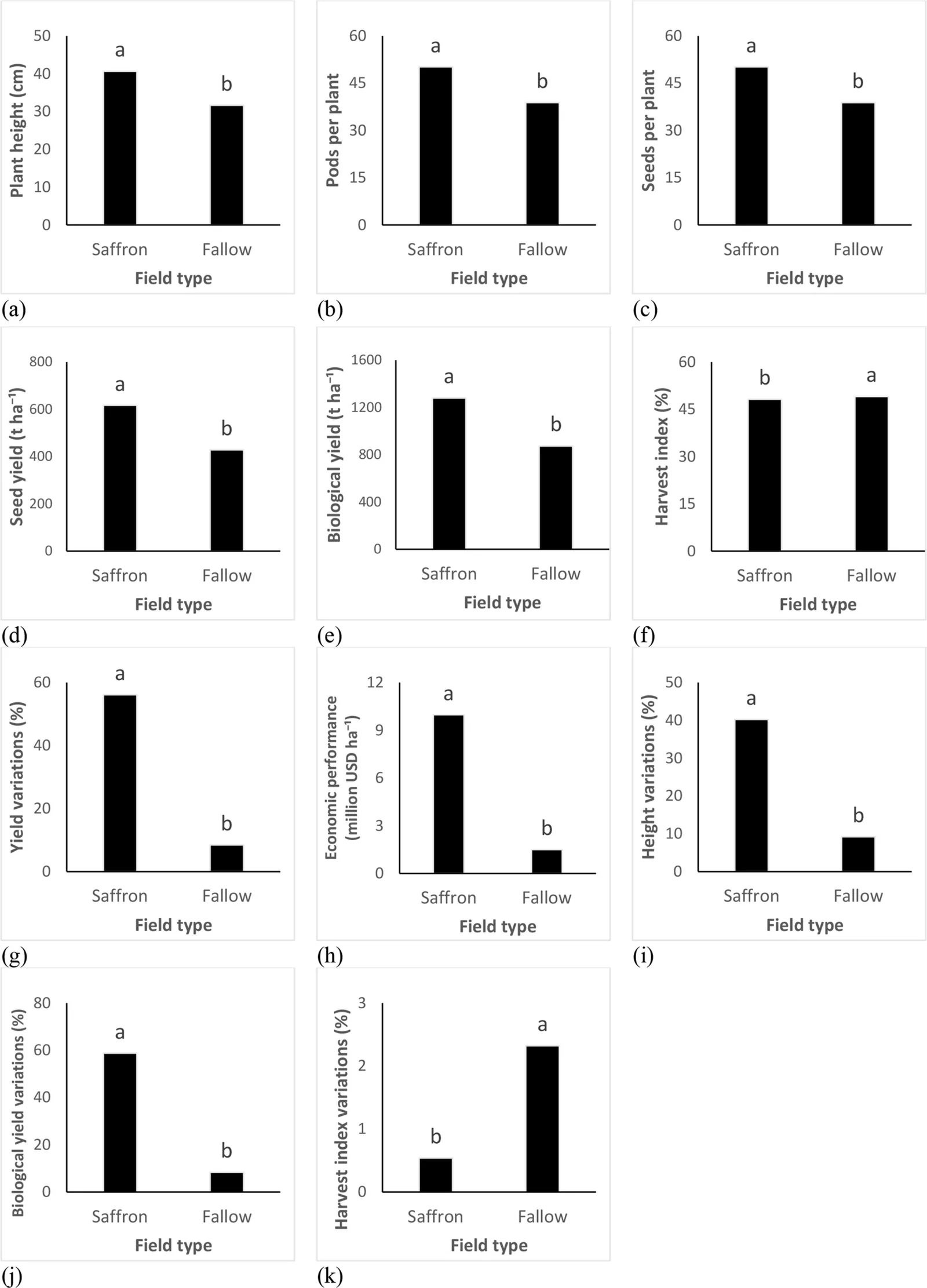
Simple and interaction effects of field and city type on morpho-physiological traits of chickpea

The mean comparison results for chickpea (Fig. 3) demonstrated that the saffron field generally outperformed the fallow field in all measured traits, except for harvest index and harvest index variation. Relative to the fallow field, cultivation in the saffron field increased plant height by 28.42%, number of pods per plant by 29.17%, number of seeds per plant by 29.17%, seed yield by 43.96%, biological yield by 46.51%, yield variation by 565.5%, economic performance by 565%, height variation by 337.6%, and biological yield variation by 603.4%, while harvest index and its variation declined by 1.73% and 76.72%, respectively.

#### The effect of field type, city type and the field ×city interaction on morphological and physiological characteristics of rapeseed

**Fig. 4:**
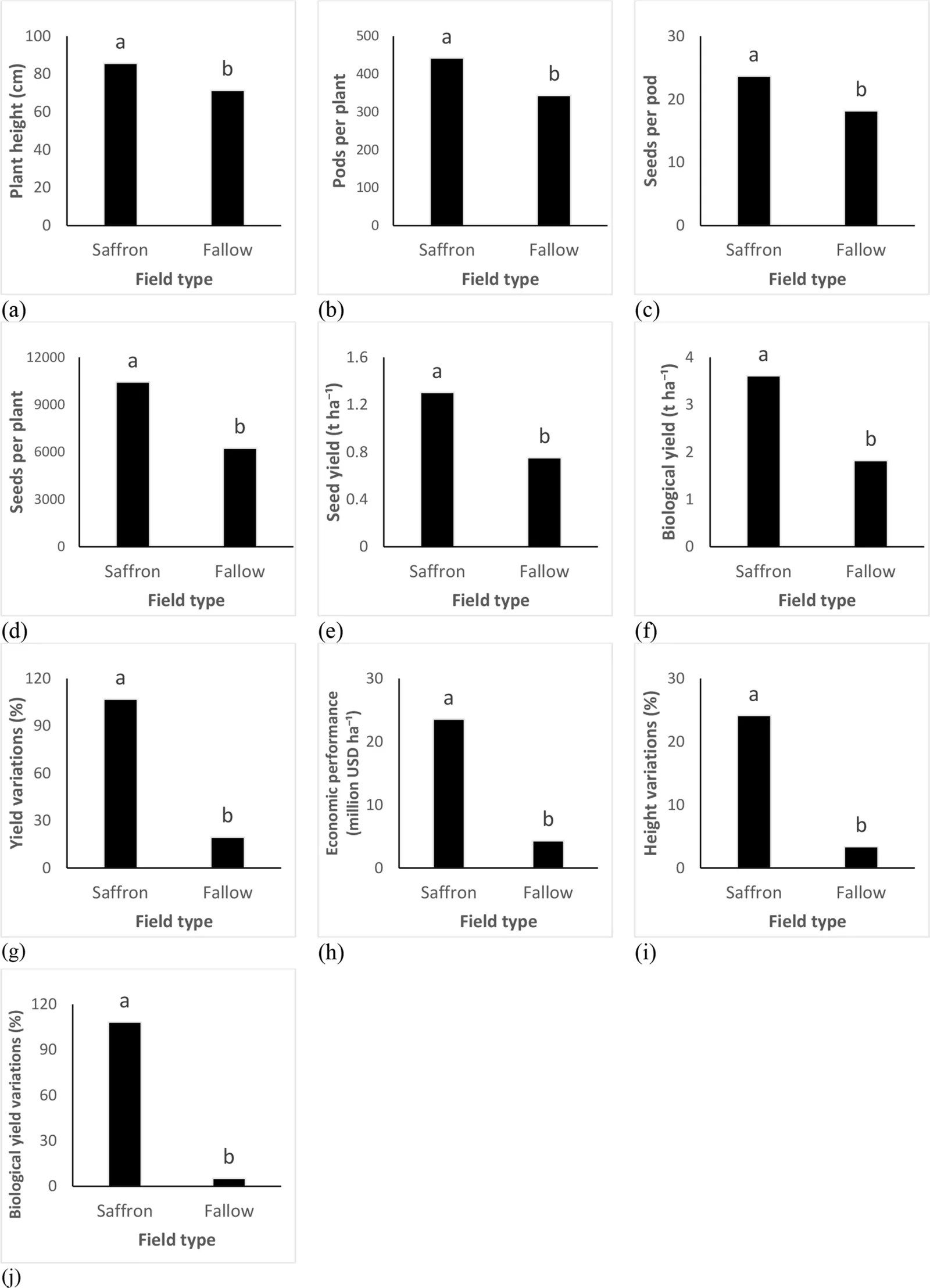
Simple and interaction effects of field and city type on morpho-physiological traits of rapeseed

As shown in Fig. 4, mean comparisons of field type for the significant traits indicated that the saffron field consistently exhibited the highest values across all traits. Compared with the fallow field, the saffron field increased plant height by 20.08%, number of pods per plant by 28.78%, number of seeds per pod by 30.28%, number of seeds per plant by 67.14%, seed yield by 73.27%, biological yield by 98.35%, yield variation by 448.4%, economic performance by 449.4%, height variation by 623.4%, and biological yield variation by 1969.5%.

#### The effect of field type, city type and interaction of field ×city on morphological and physiological characteristics of sesame

**Fig. 5:**
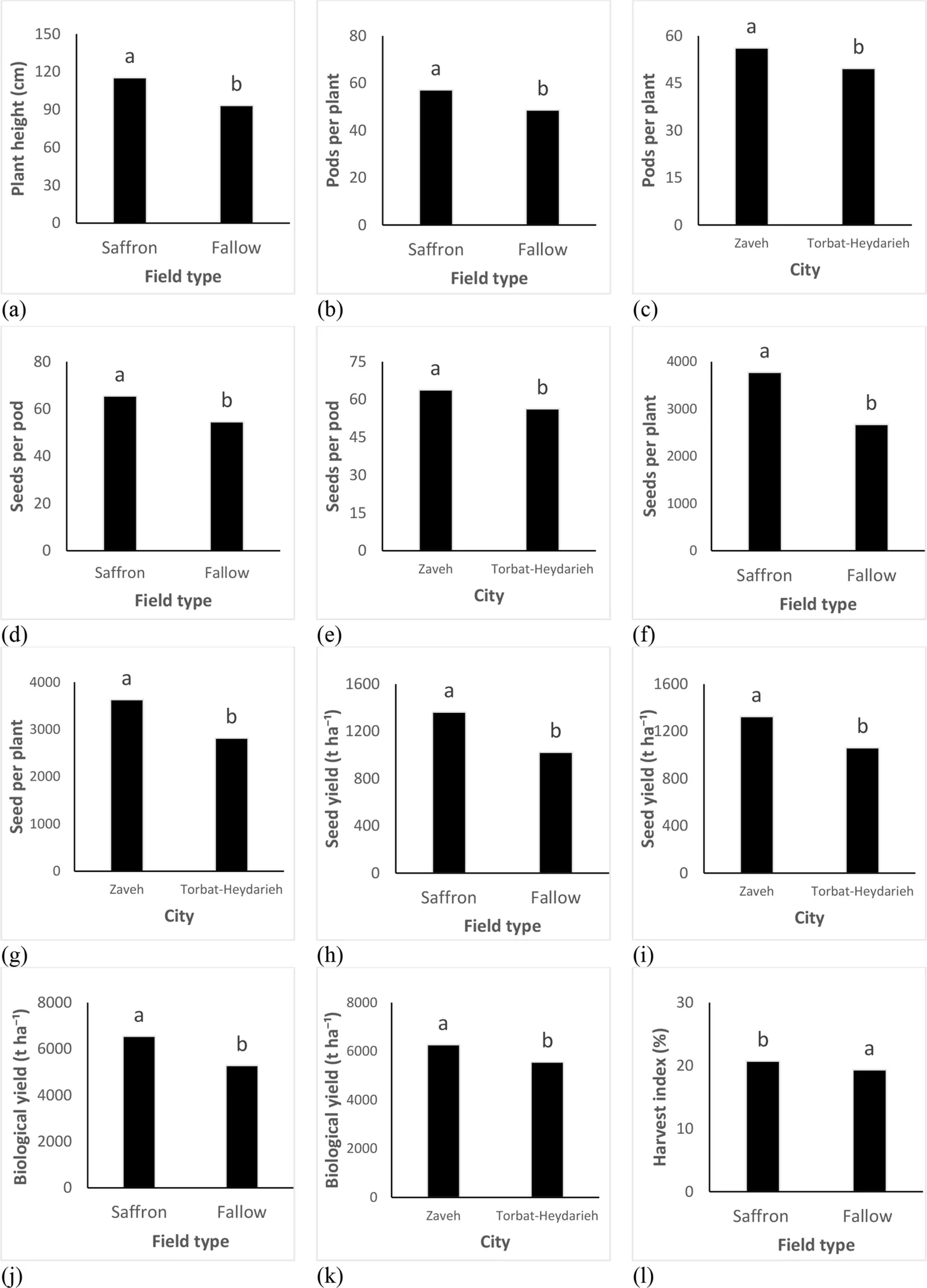

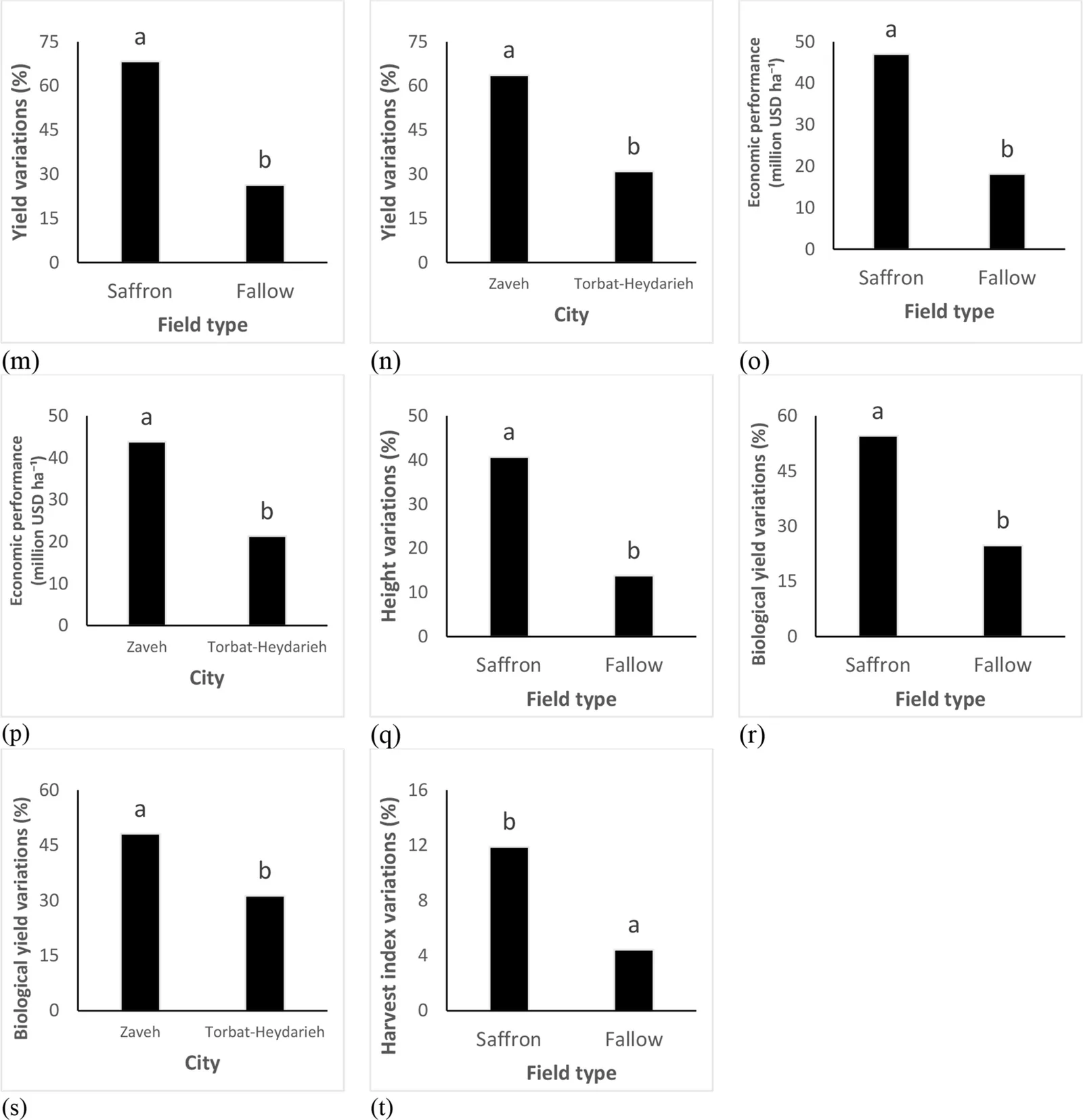
Simple and interaction effects of field and city type on morpho-physiological traits of sesame

The mean comparison results shown in Fig. 5 indicated that the saffron field under the field type treatment and Zaveh under the city treatment exhibited the highest values for all significant traits. Under the field type treatment, cultivation in the saffron field increased plant height (23.57%), number of pods per plant (17.46%), number of seeds per pod (19.96%), number of seeds per plant (41.24%), seed yield (33.26%), biological yield (23.87%), harvest index (7.13%), yield variation (159.83%), economic performance (159.76%), height variation (194.13%), biological yield variation (120.26%), and harvest index variation (168.5%). Similarly, under the city type treatment, Zaveh showed higher values than Torbat Heydarieh for all significant traits, with increases of 13.08% in number of pods per plant, 13.31% in number of seeds per pod, 28.90% in number of seeds per plant, 24.94% in seed yield, 12.83% in biological yield, 105.55% in yield variation, 105.48% in economic performance, and 53.93% in biological yield variation.

#### Combined analysis of field type, cultivation city, and crop species on different performance components in five crops

The combined analysis of variance (Table 8) indicated that economic performance and harvest index variations were significant at the 0.01 probability level among crops. Yield and biological yield were significant at the 0.05 level, whereas plant height did not show a significant difference among crops.

**Table 8:** ANOVA analysis of different performance components parameters in five crops

Sources of Variation	df	Yield variations	Economic performance	Height variations	Biological yield variations	Harvest index variations
Block	2	48	713.32	225.1	221	21.68
Crop	4	1795^*^	15,353^**^	625.2^ns^	1520^*^	2060.64^**^
Ea	8	430	953.83	188.4	349	15.77
City	1	3531^**^	6504^**^	1083.9^**^	3087^**^	23.24^ns^
City × Crop	4	1016^**^	3471.2^**^	306.9^ns^	367^*^	104.6^ns^
Eb	10	131	130.73	91.3	62	49.12
Field	1	42,054^**^	8819.7^**^	10,861.8^**^	47,380^**^	189.57^ns^
Field × Crop	4	1952^**^	698.78^**^	60.5^ns^	3205^**^	237.06^**^
Ec	20	234	108.24	68	179	52.08

^*^significance at *P* < 0.05, ^**^significance at *P* < 0.01, ^ns^ no significance

ANOVA results also showed that between different cities, except for the attribute of harvest index variations, which did not show a significant difference, significant differences were observed in other attributes at the 0.01 level.

The interaction effect of city × plant also showed significant differences at the 0.01 level for yield variations and economic performance, and at the 0.05 level for biological yield variations. However, no significant differences were observed for height variations and harvest index variations. As shown in Table 8, significant differences among different fields were observed at the 0.01 probability level for all traits, except for harvest index variations, which did not show a significant difference. Also, in the Field × crop interaction effect, significant differences were observed at the 0.01 level for all traits, except for height variations, which did not show a significant difference. In contrast, the interaction effects of City × Field and City × Field × crop were not significant for any of the evaluated traits.

### Biological yield variations

**Fig. 6:**
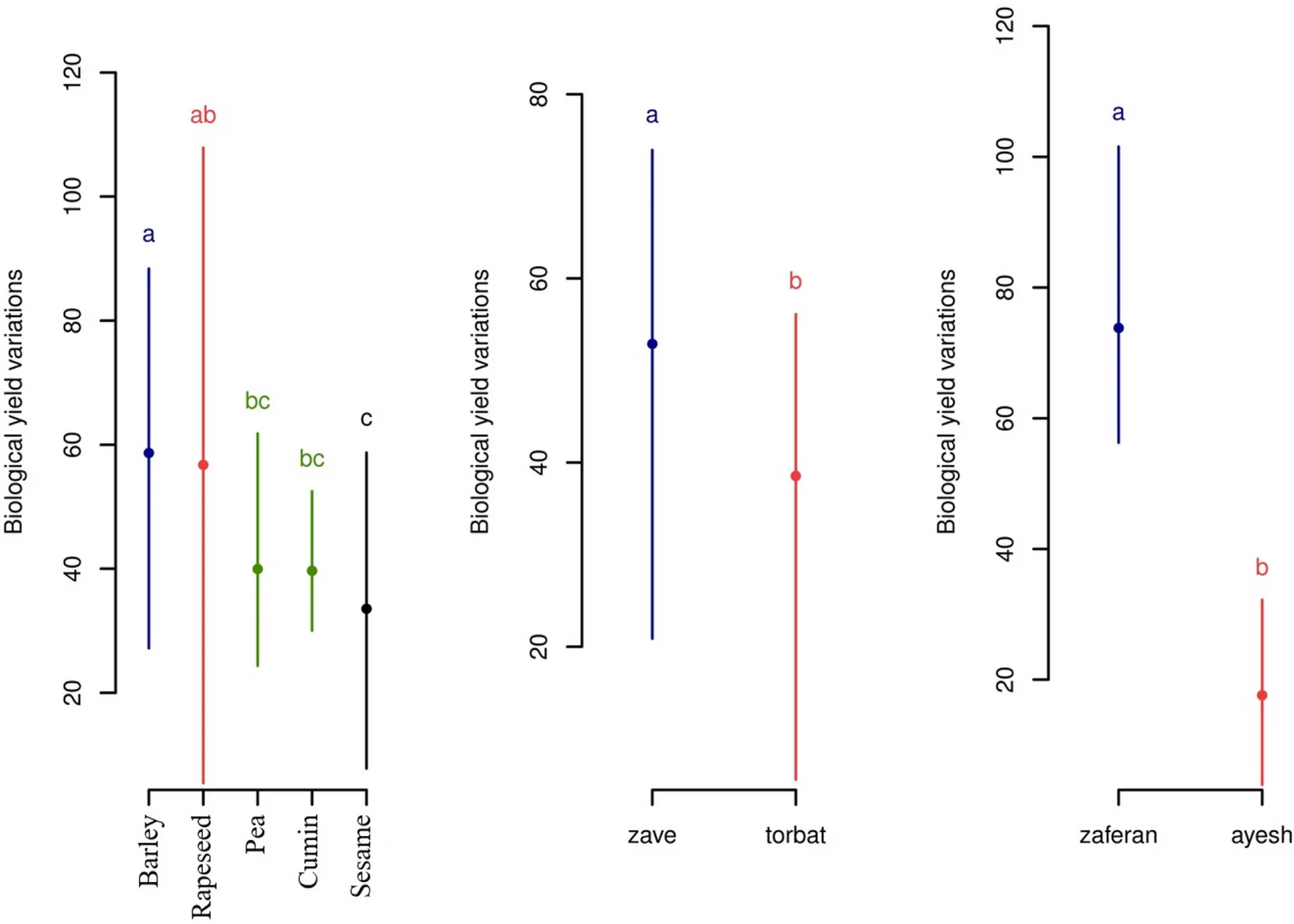
Effects of crop species, city, and field type on biological yield variation

According to the mean comparison results in Fig. 6, the highest biological yield variations were observed in barley (58.66%), followed by rapeseed (56.73%), while the lowest value was recorded in chickpea (33.55%). Cumin (39.96%) and sesame (39.67%) exhibited similar levels of biological yield variations. The average biological yield variations in Zaveh (52.89%) were higher than those in Torbat-Heydarieh (38.54%). Also, the average biological yield variations in the saffron field (73.81%) were significantly higher than those observed in the fallow field (17.61%).

#### Economic performance variations

**Fig. 7:**
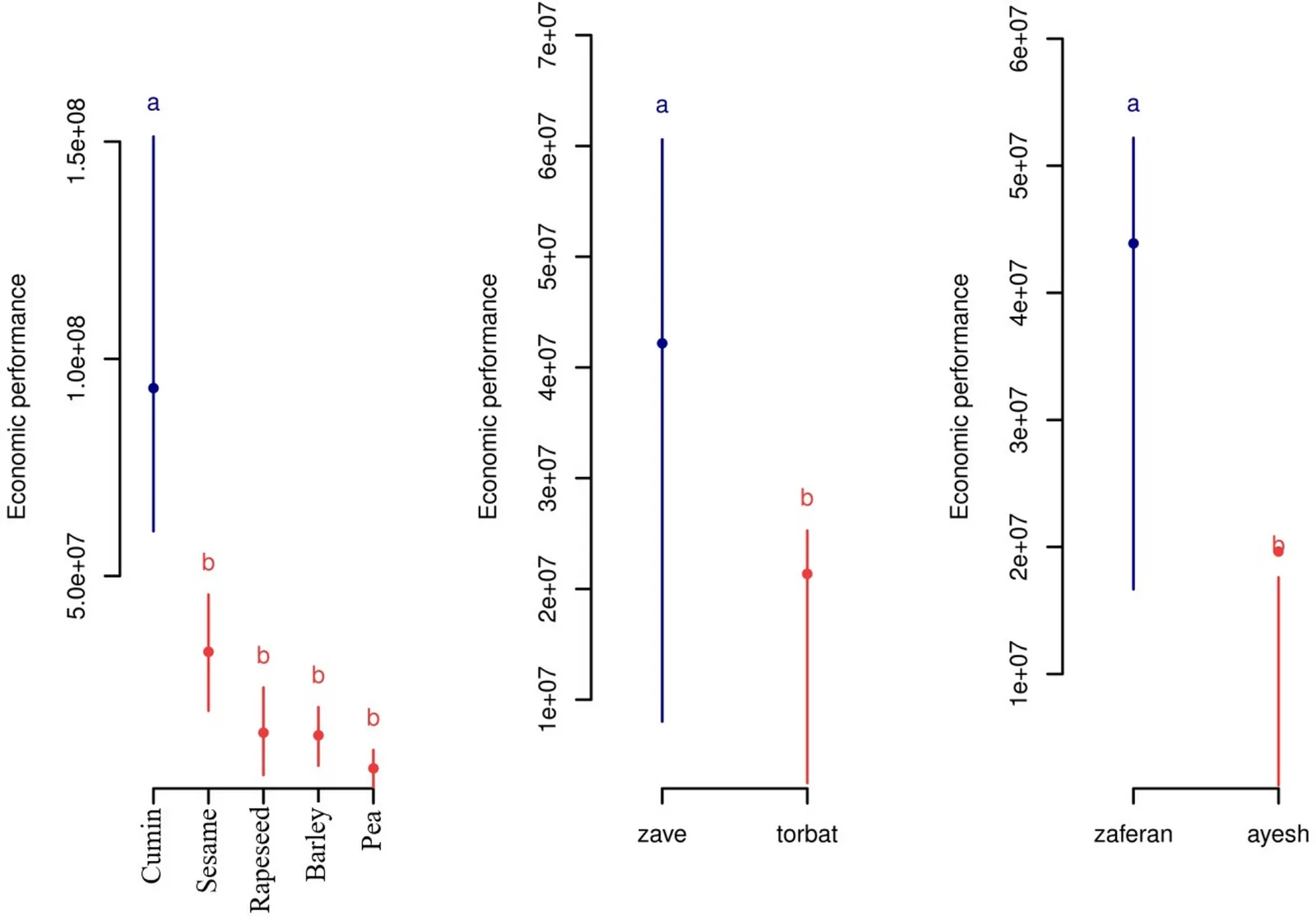
The combined effects of city, field and plant type on economic performance

The mean comparison results presented in Fig. 7 indicated that cumin (93.26%) exhibited the highest economic performance among the five crops. In contrast, sesame (32.56%), rapeseed (13.93%), barley (13.33%), and chickpea (5.73%) did not differ significantly in terms of economic performance. Furthermore, Zaveh (42.17%) showed a significantly higher economic performance than Torbat Heydarieh (21.35%). In addition, the saffron field (43.89%) exhibited a higher economic performance compared with the fallow field (19.64%).

#### Harvest index variation

**Fig. 8:**
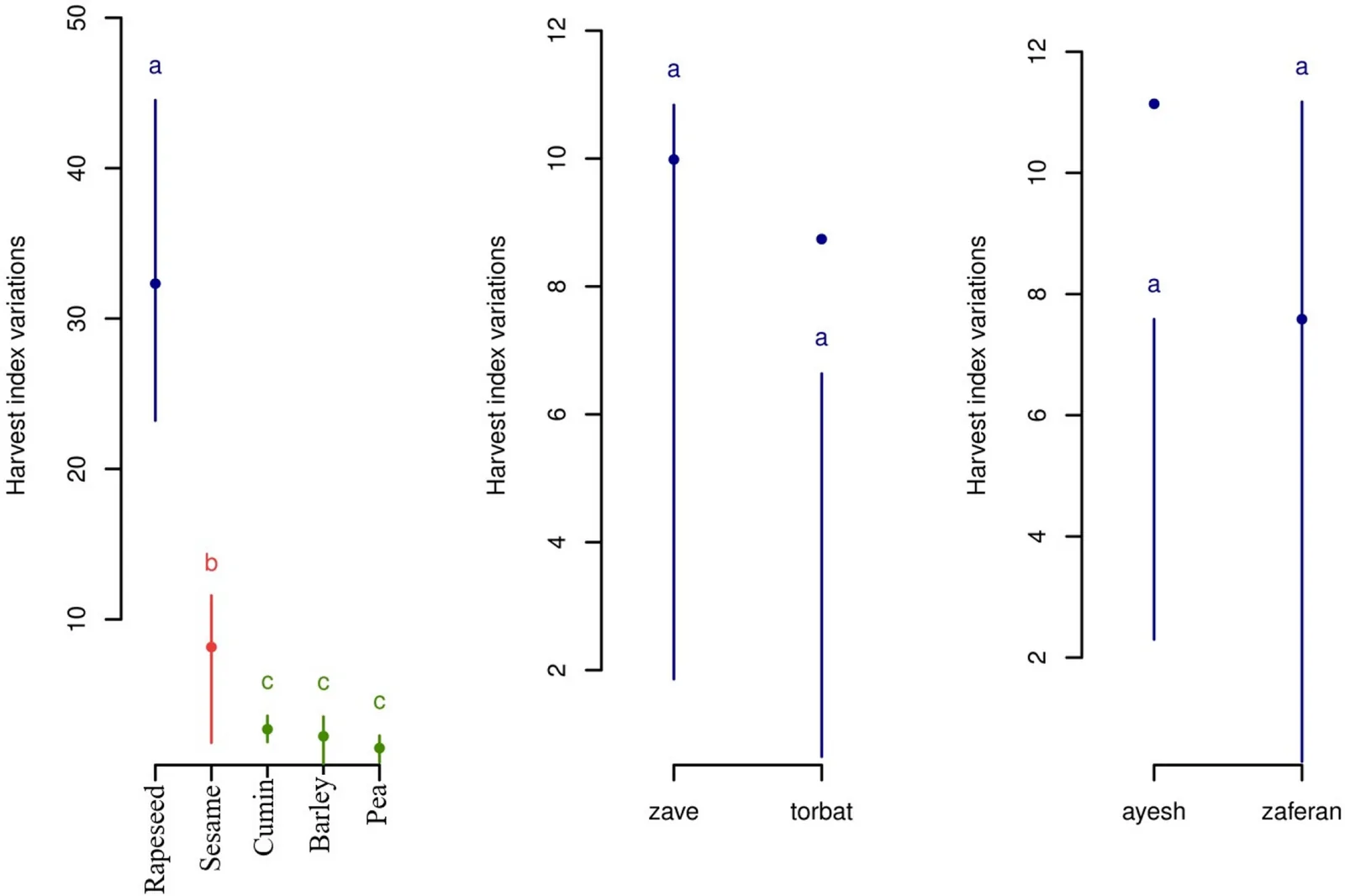
The combined effects of city, field and plant type on harvest index variation

According to Fig. 8, rapeseed exhibited the highest harvest index variation (32.31%), followed by sesame (8.15%). In contrast, cumin (2.68%), barley (2.21%) and chickpea (1.43%) showed relatively low harvest index variation, with no significant differences among them. Furthermore, no significant differences in harvest index variation were observed between the cities of Zaveh (9.98%) and Torbat-Heydarieh (8.74%), nor between the saffron fields (11.13%) and fallow (7.58%).

#### Height variations

**Fig. 9:**
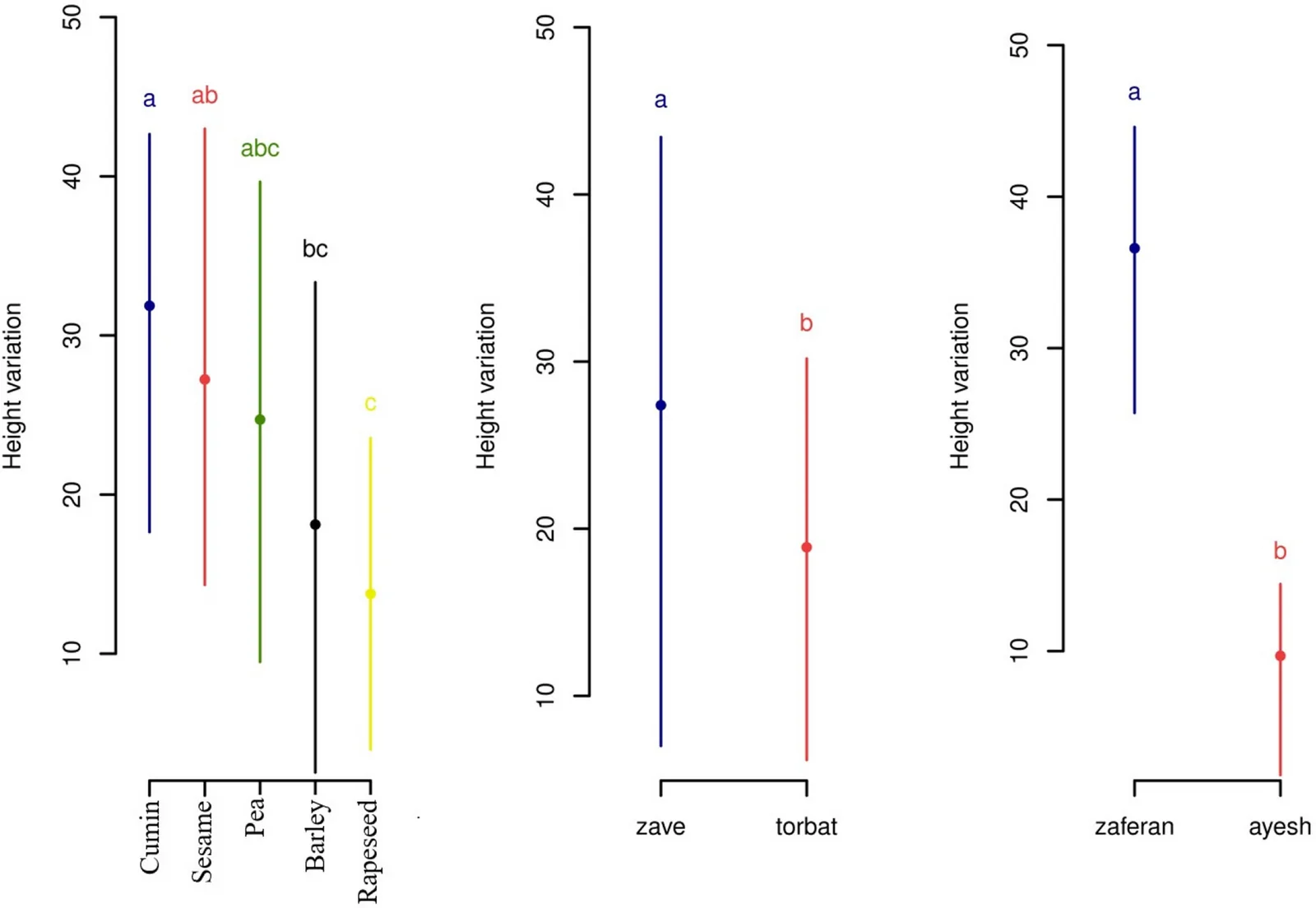
The combined effects of city, field and plant type on height variations

The mean comparison results shown in Fig. 9 indicated that cumin exhibited the highest height variation (31.86%), followed by sesame (27.23%), chickpea (24.71%), and barley (18.11%), while rapeseed showed the lowest height variation (13.76%). Furthermore, greater height variation was observed in Zaveh (27.38%) than in Torbat Heydarieh (18.88%), as well as in the saffron field (36.59%) compared with the fallow (9.68%).

#### Yield variations

**Fig. 10:**
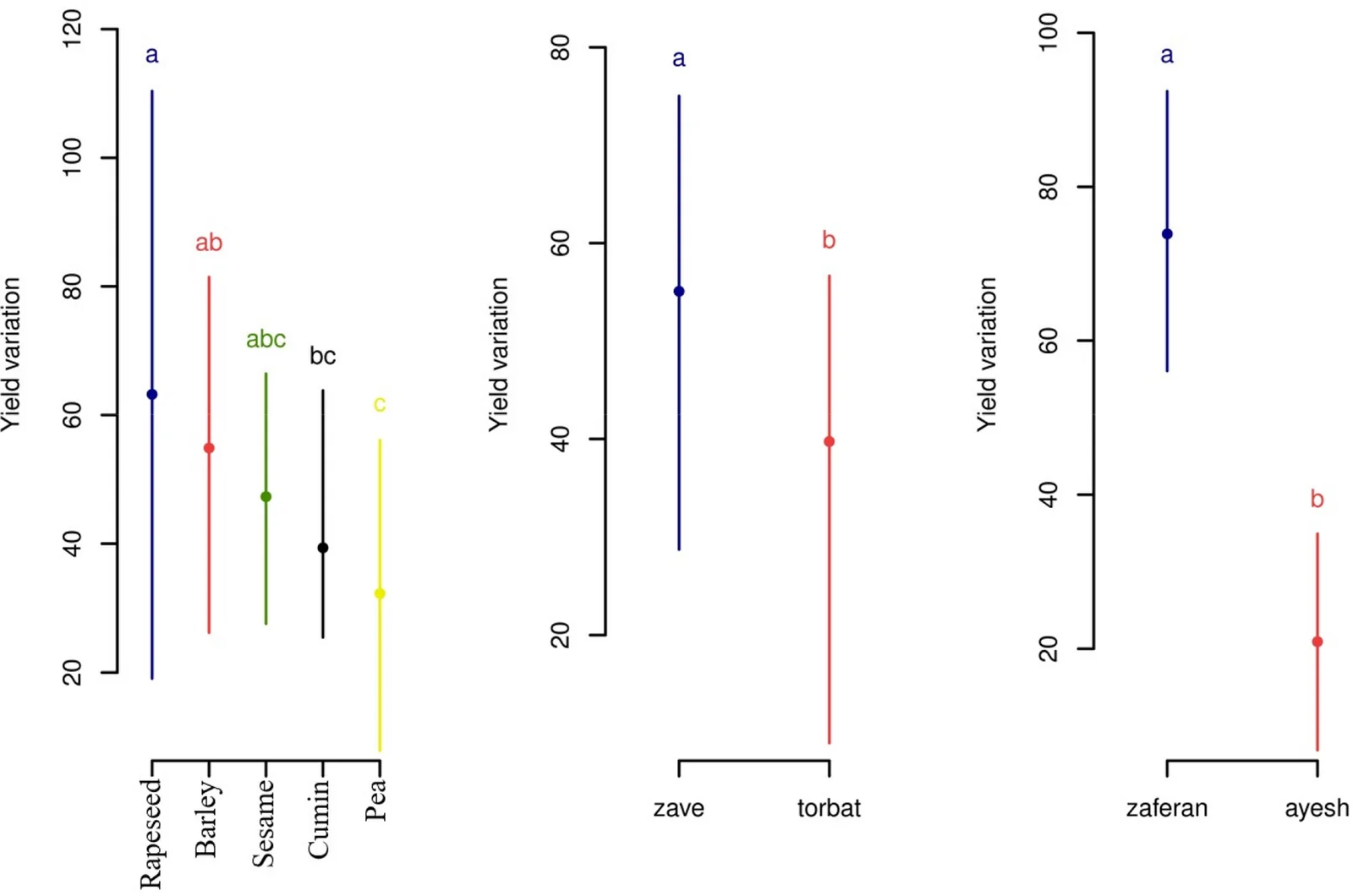
The combined effects of city, field and plant type on yield variations

The mean comparison analysis in Fig. 10 showed that the highest yield variations were observed in rapeseed (63.20%), followed by barley (54.85%), sesame (47.30%), and cumin (39.37%), while chickpea exhibited the lowest yield variations (32.27%). On the other hand, yield variations were higher in Zaveh (55.07%) than in Torbet-Heydarieh (39.73%), and in the saffron field (73.87%) compared with the fallow (20.92%).

### Soil–crop trait correlation analysis

**Fig. 11:**
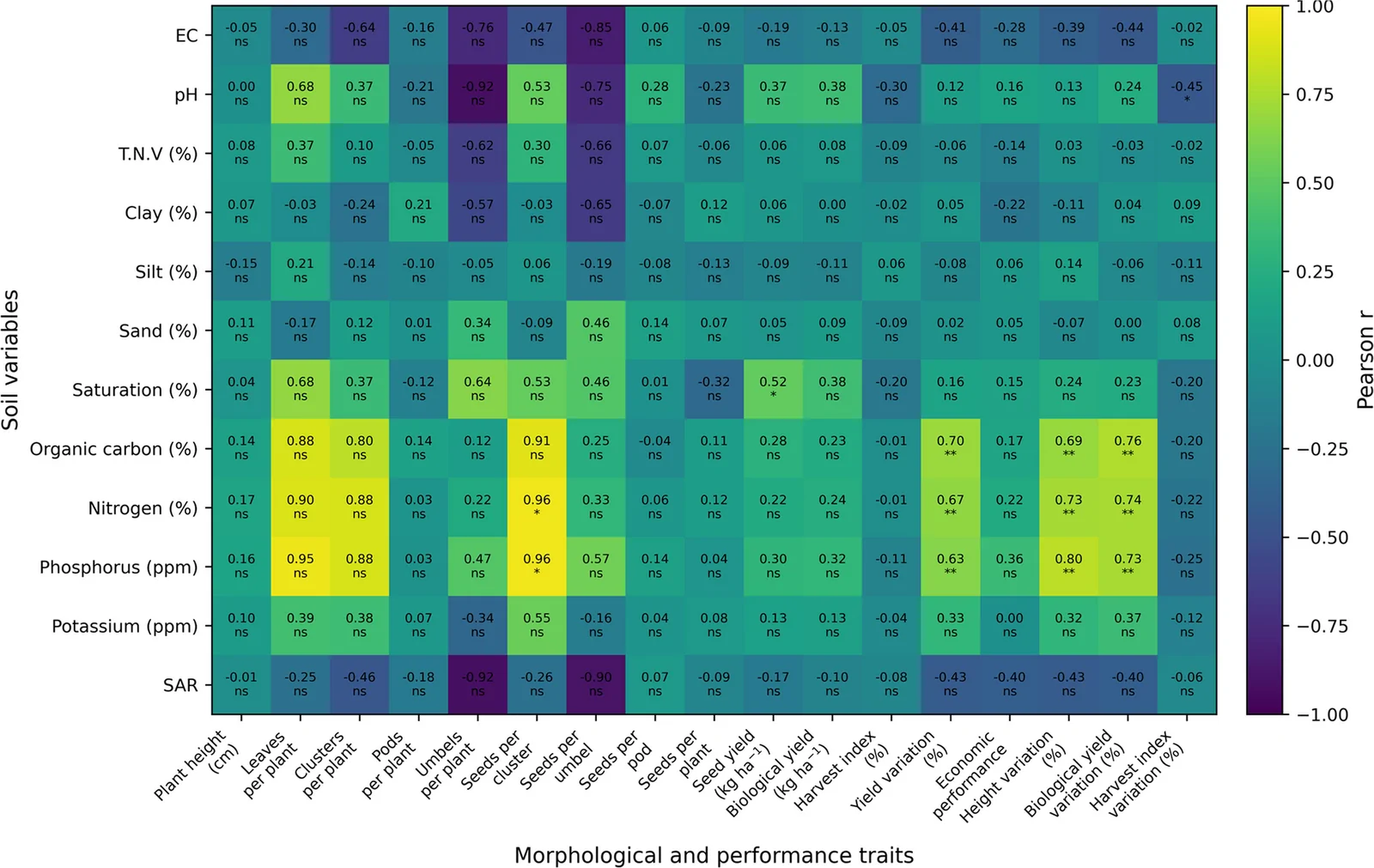
Pearson correlation heatmap between post-cultivation soil properties and crop morpho-physiological and performance-related traits. Values inside cells represent Pearson correlation coefficients (r). Significance levels are indicated as *P* < 0.05 (*) and *P* < 0.01 (**); ns, not significant

Correlation analysis was performed to examine the association between post-cultivation soil properties and crop morpho-physiological and performance-related traits (Fig. 11). Overall, most direct morphological traits, including plant height, pods per plant, seeds per pod, seeds per plant, seed yield, harvest index, and economic performance, did not show significant correlations with the measured soil variables. However, several soil fertility-related variables were significantly associated with derived yield-response indices. Organic carbon showed significant positive correlations with yield variation (r = 0.70, *P* < 0.01), height variation (r = 0.69, *P* < 0.01), and biological yield variation (r = 0.76, *P* < 0.01). Similarly, nitrogen was positively correlated with yield variation (r = 0.67, *P* < 0.01), height variation (r = 0.73, *P* < 0.01), and biological yield variation (r = 0.74, *P* < 0.01). Phosphorus also showed significant positive correlations with yield variation (r = 0.63, *P* < 0.01), height variation (r = 0.80, *P* < 0.01), and biological yield variation (r = 0.73, *P* < 0.01).

Among the direct yield traits, biological yield showed a significant positive correlation with soil saturation percentage (r = 0.52, *P* < 0.05), whereas harvest index variation was negatively correlated with soil pH (r = − 0.45, *P* < 0.05). EC, T.N.V., soil texture fractions, potassium, and SAR did not show consistent significant correlations with most crop traits. Overall, organic carbon, nitrogen, and phosphorus were the soil variables most closely associated with relative changes in crop performance, particularly yield variation, height variation, and biological yield variation.

## Discussion

This study evaluated how field history and location influence the growth, yield, and economic performance of five subsequent crops, including barley, cumin, chickpea, rapeseed, and sesame, in two semi-arid saffron-producing regions of Iran. The main scientific contribution of this study is that it examines the residual effect of long-term saffron cultivation on subsequent crop performance compared with fallow management, an issue that has received limited attention in saffron-based production systems. This is particularly important because water scarcity is a major constraint in Iran’s arid and semi-arid regions, and improving resource-use efficiency in saffron-based systems has been emphasized in recent studies on saffron intercropping and irrigation management [13]. Moreover, recent evidence shows that crop rotation can improve soil microbial biomass carbon and nitrogen compared with monoculture systems, indicating that previous crop history may influence nutrient cycling and subsequent crop performance [6]. In this context, replacing or reducing fallow periods should be based on suitable crop selection, because fallow replacement strategies are not universally beneficial and must be adapted to soil water and nutrient conditions in semi-arid environments [14]. Therefore, the present study provides field-based evidence on whether post-saffron fields can be used as productive alternatives to fallow land. The discussion interprets the results in relation to soil fertility status, crop-specific responses, location-dependent effects, and market-based economic performance, with emphasis on how post-saffron cultivation can contribute to sustainable land management and integrated soil fertility management in semi-arid agroecosystems. Integrated soil fertility management is particularly relevant here because it combines crop choice, nutrient management, organic inputs, and site-specific soil management to improve crop productivity under semi-arid conditions [15, 16].

### Soil composition and plant performance

The soil results indicate that differences in crop performance were associated with the initial fertility and salinity status of the two field types and locations. In Torbat Heydarieh, both soils were slightly alkaline and calcareous; however, the fallow field had higher EC and SAR and lower organic carbon, nitrogen, phosphorus, and potassium than the post-saffron field. These conditions suggest that the fallow soil was more affected by salinity and had weaker nutrient availability, which may have limited crop growth and yield formation. This interpretation is supported by recent evidence showing that salt-affected soils reduce crop productivity by limiting water uptake, inducing osmotic and ionic stress, and disrupting nutrient availability, particularly in arid and semi-arid regions [17, 18].

In Zaveh, soils had lower EC values than those in Torbat Heydarieh, indicating more favorable non-saline conditions for crop establishment and growth. The post-saffron soil in Zaveh also had higher organic carbon and NPK contents than the fallow soil, suggesting that previous long-term saffron cultivation may have created a more favorable soil fertility legacy for subsequent crops. Such legacy effects may be associated with long-term field management, residual root biomass, nutrient inputs, and changes in soil biological activity. Recent studies have shown that diversified crop rotations can improve soil microbial diversity, organic carbon dynamics, and nutrient cycling, all of which are important indicators of soil health and sustainable crop production [19–21].

After cultivation, most soil chemical properties showed only limited changes compared with the initial values, especially in Torbat Heydarieh. Therefore, the better performance of crops in post-saffron fields was more likely related to pre-existing soil fertility conditions and field history rather than short-term changes during a single growing season. In addition, apparent changes in clay, silt, and sand percentages should be interpreted cautiously, because soil texture is a relatively stable property and short-term differences may reflect spatial variability or sampling effects rather than actual texture modification by crop growth. Overall, the higher organic carbon and nutrient status and the lower salinity of post-saffron soils, particularly in Zaveh, were consistent with the improved growth, yield, and economic performance of subsequent crops compared with fallow fields.

The soil–crop trait correlation analysis provided quantitative support for the relationship between soil fertility status and crop responses. Among the measured soil properties, organic carbon, nitrogen, and phosphorus showed the strongest positive correlations with relative crop-response indices, particularly yield variation, height variation, and biological yield variation. Organic carbon was positively correlated with yield variation, height variation, and biological yield variation, and similar positive correlations were observed for nitrogen and phosphorus. Biological yield was also positively correlated with soil saturation percentage, whereas harvest index variation showed a negative correlation with soil pH. These results indicate that organic carbon, nitrogen, and phosphorus were the soil variables most closely linked with the improved relative performance of crops grown in post-saffron fields.

### The influence of field type on crop traits

The differences in crop performance between post-saffron and fallow fields indicate that previous field history and associated management conditions can strongly influence the growth and productivity of subsequent crops. In this study, post-saffron fields generally produced better responses in important traits such as seed yield, biological yield, and plant height, although the magnitude of response varied among crop species. These responses were linked not only to the current growing season, but also to the contrasting land-use histories of the two field types. Long-term saffron cultivation may have contributed to residual root biomass, organic inputs, nutrient cycling, and changes in soil biological activity, whereas the fallow fields were affected by the absence of crop production, shallow tillage, weed biomass incorporation, and differences in weed pressure. These management-related differences were reflected in soil fertility status and may have influenced the performance of the subsequent crops.

The superior performance of crops in post-saffron fields was consistent with the relatively better soil fertility status observed in these fields, particularly higher organic carbon and NPK contents compared with fallow fields. Previous studies have shown that fields with a history of diversified cropping or rotation can benefit from improved soil organic matter decomposition, greater nutrient availability, better soil structure, and enhanced microbial activity [22]. Similarly, diversified crop rotations have been reported to improve crop productivity and soil health by increasing soil organic carbon and stimulating microbial activity [23],Ghanbari Moheb [24]. Therefore, the better performance of barley, rapeseed, sesame, cumin, and chickpea in post-saffron fields may reflect the combined influence of land-use history, residual organic inputs, weed-related management, improved nutrient status, and crop-specific responsiveness.

The crop-specific responses also provide an important insight. Rapeseed and barley showed strong improvements in seed and biological yield, which may be related to their high nutrient demand and their ability to respond to improved soil fertility. In contrast, cumin and sesame are more adapted to warm and dry environments, and their better performance in post-saffron fields may reflect both improved soil conditions and their suitability for semi-arid saffron-producing areas. This is particularly important from a practical perspective, because crop selection after saffron should not be based only on biological yield, but also on adaptation, market value, and economic return.

Another possible explanation for the positive effect of post-saffron fields is related to rhizosphere processes. Long-term perennial cultivation can influence soil microbial communities through root residues, rhizodeposition, and repeated field management. Recent studies have shown that crop rotation and crop diversity can increase microbial biomass and support soil functions related to nutrient cycling [25, 26]. In saffron-based systems, microbial interactions have also been shown to affect soil chemical properties and rhizosphere microbial communities, which may influence plant growth and productivity [27]. Although microbial activity was not directly measured in the present study, the observed improvement in crop performance in post-saffron fields suggests that future research should examine microbial and biochemical soil indicators to better explain the mechanisms behind the residual effect of saffron cultivation.

Overall, the field-type effect indicates that post-saffron fields can provide a more favorable environment for subsequent crops than fallow fields, especially when suitable crop species are selected. This finding adds a practical insight for semi-arid saffron-producing regions: land after saffron cultivation should not necessarily remain fallow, but can be used for selected crops that combine agronomic adaptation with economic value.

### City-specific effects and crop performance

The comparison between Torbat Heydarieh and Zaveh indicates that local agroecological conditions affected crop performance in a crop- and trait-specific manner, rather than uniformly across all species. Although both regions are located in semi-arid saffron-producing areas, the soil data showed that Zaveh had generally lower EC values and more favorable non-saline conditions than Torbat Heydarieh. This difference is important because salinity and sodicity can restrict crop growth by reducing water uptake, disturbing nutrient balance, and limiting physiological processes related to yield formation [18, 28]. Therefore, where significant city effects were observed, particularly for cumin and sesame, the better performance in Zaveh was consistent with its more favorable soil conditions.

The city effect was not uniform for all crops, indicating that crop response depended on species-specific adaptation and local environmental conditions. Cumin and sesame showed clearer advantages in Zaveh, which may be related to their adaptation to warm and dry environments and their ability to benefit from lower soil salinity and improved nutrient availability. This supports the idea that crop selection in semi-arid regions should be site-specific rather than generalized, because soil fertility, salinity status, and climatic conditions jointly determine crop suitability and productivity. Recent studies also emphasize that assessing soil fertility and site-specific soil variability is essential for improving nutrient management and sustainable crop production in semi-arid agroecosystems [29].

Another important point is that the positive effect of post-saffron fields was generally observed across both locations, while the city × field type interaction was not consistently significant for most traits. This suggests that the advantage of post-saffron cultivation over fallow management was relatively stable across the two locations, and any numerical differences in magnitude should be interpreted descriptively rather than as statistically confirmed interaction effects. In this regard, Zaveh provided more favorable background soil conditions; however, the residual benefit of post-saffron cultivation was not statistically dependent on location for most traits. This finding is consistent with broader evidence showing that crop yield responses are strongly influenced by local climate, soil properties, and spatial heterogeneity, and that these factors should be considered when interpreting field-level productivity differences [30, 31].

Overall, the city-specific results provide a practical insight for the study area: post-saffron cultivation can be recommended as a productive alternative to fallow management in both regions, but crop choice should be adjusted to local soil and environmental conditions. In Zaveh, where salinity was lower and soil conditions were more favorable, cumin and sesame showed clearer city-related advantages, while rapeseed showed strong potential mainly because of its response to post-saffron field conditions. In Torbat Heydarieh, where salinity was relatively higher, post-saffron cultivation should be accompanied by improved soil fertility management, salinity control, and careful crop selection to achieve stable productivity.

### Economic and yield performance analysis

The economic performance of the studied crops provided an important practical dimension to the agronomic results. In this study, cumin showed the highest market-based economic performance, followed by sesame and rapeseed. This pattern indicates that crop selection after saffron cultivation should not be based only on biological yield or seed yield, but also on market value, local demand, and the ability of each crop to generate income under semi-arid conditions. Cumin is a high-value spice crop that is commonly cultivated in arid and semi-arid environments, and recent studies have highlighted the economic importance of cumin-based cropping systems and their potential to improve land-use efficiency and farm returns [32].

The higher economic performance of sesame and rapeseed also reflects the importance of oilseed crops in semi-arid agricultural systems. Sesame is particularly relevant for Iran because it is widely cultivated in warm and dry regions, has relatively lower water requirements than several other oilseed crops, and has strong domestic market importance [33]. Therefore, the better performance of sesame in post-saffron fields suggests that this crop may be a suitable option for farmers seeking both agronomic adaptation and economic return after saffron cultivation. Rapeseed also showed strong yield response in post-saffron fields, indicating that crops with higher nutrient demand may benefit from the relatively better fertility status of these fields.

In contrast, barley and chickpea showed lower economic performance in this study, which may be related to their lower market-based return under the price structure used for the 2022–2023 growing season. This does not necessarily mean that these crops are unsuitable for the region; rather, it shows that their value in a post-saffron system may be more related to food security, soil improvement, or rotational benefits than short-term economic return alone. For example, legumes such as chickpea can contribute to soil fertility through biological nitrogen fixation, while cereals such as barley can provide stable production under semi-arid conditions. Therefore, economic interpretation should be combined with agronomic and soil fertility considerations.

The comparison between field types also showed that post-saffron fields created better conditions for economic performance than fallow fields. This result supports the idea that productive use of post-saffron land can increase land-use efficiency and reduce the opportunity cost of leaving land fallow. Similar evidence from integrated crop management studies has shown that improved soil and nutrient management can increase crop yield and farm income across cereals, legumes, and oilseeds [34]. Thus, the economic advantage observed in post-saffron fields should be interpreted as a combined outcome of crop yield response, crop market price, soil fertility status, and local environmental conditions.

Overall, the economic results provide a useful recommendation for the study area. In Zaveh, where soil conditions were more favorable, cumin, sesame, and rapeseed appear to be promising options for improving farm income after saffron cultivation. In Torbat Heydarieh, where salinity was relatively higher, economic gains from post-saffron cultivation may require more careful soil fertility management, salinity control, and crop selection. These findings suggest that post-saffron crop planning should integrate both yield performance and market-based economic value to support sustainable and profitable land use in semi-arid saffron-producing regions.

### Variations in growth traits

The variation indices used in this study provide useful information about the relative magnitude of crop responses to field type and location. These indices show how strongly each crop responded to post-saffron cultivation or fallow management and help compare the intensity of responses among crop species. The larger variation values observed for seed yield, biological yield, and plant height in some crops indicate greater sensitivity to changes in soil fertility, salinity status, and local environmental conditions.

Rapeseed and barley showed relatively high variation in yield-related traits, particularly in post-saffron fields. This suggests that these crops were more responsive to the improved soil fertility conditions observed after saffron cultivation. Such responses are agronomically reasonable because biological yield and plant height are closely linked to biomass accumulation, nutrient uptake, and canopy development, while seed yield depends on the successful conversion of vegetative growth into reproductive output. Recent studies have shown that crop rotation can improve soil quality, increase microbial biomass carbon and nitrogen, and enhance yield formation in barley-based systems [35]. Similarly, sustainable soil health practices can improve crop growth and productivity by maintaining soil physical, chemical, and biological functions [36, 37].

The response of harvest index was weaker and more crop-specific than seed yield or biological yield. This indicates that post-saffron fields mainly improved total biomass production and seed yield, while biomass partitioning to the economic yield was not equally improved in all crops. Harvest index reflects the efficiency of converting above-ground biomass into harvestable yield and is strongly influenced by crop genotype, environmental conditions, and biomass allocation patterns [38]. Therefore, the lower or limited change in harvest index for some crops does not necessarily contradict the positive effect of post-saffron cultivation,rather, it suggests that improved soil conditions increased crop growth and biomass production more strongly than the partitioning efficiency of biomass into grain or seed.

The observed differences among crops also highlight the importance of crop-specific adaptation. Rapeseed responded strongly in seed and biological yield, which may reflect its high nutrient demand and strong response to improved fertility. Barley also showed a clear response, consistent with evidence that barley yield and biomass can be strongly affected by previous crop history and soil nutrient conditions [39]. In contrast, crops such as cumin and sesame may show more moderate variation because they are naturally better adapted to warm and dry environments and may maintain performance under less favorable conditions. Overall, the variation indices suggest that post-saffron cultivation did not affect all crops in the same way,instead, it enhanced crop performance according to each species’ nutrient demand, stress tolerance, and capacity for biomass accumulation and yield formation.

### Implications for sustainable agriculture

The results of this study highlight the potential role of post-saffron field management in improving the productivity of subsequent crops in arid and semi-arid regions. This conclusion is supported by the higher seed yield observed in post-saffron fields compared with fallow fields, with increases of 55.7% in barley, 15.75% in cumin, 43.96% in chickpea, 73.27% in rapeseed, and 33.26% in sesame. Biological yield also improved in post-saffron fields, ranging from an 18.71% increase in cumin to a 98.35% increase in rapeseed. These results indicate that fields previously cultivated with saffron provided more favorable conditions for biomass production and yield formation than fallow fields.

From a sustainable agriculture perspective, these findings suggest that post-saffron fields can reduce the need for non-productive fallow periods and improve land-use efficiency when suitable crops are selected. The economic results further support this interpretation, as market-based economic performance was higher in post-saffron fields, with cumin showing the highest economic performance, followed by sesame and rapeseed. Therefore, these crops may be considered promising options after saffron cultivation in the study area because they combine agronomic adaptation with economic value.

The soil results also justify linking this practice to integrated soil fertility management. Post-saffron fields generally had better fertility status than fallow fields, particularly in terms of organic carbon and NPK contents, while Zaveh showed lower salinity and more favorable soil conditions than Torbat Heydarieh. Therefore, using suitable crops after saffron cultivation should be combined with soil testing, balanced fertilization, organic matter conservation, salinity management, and site-specific crop selection. Overall, the findings suggest that post-saffron cultivation may contribute to improving land-use efficiency and supporting subsequent crop production in the studied regions, particularly when combined with appropriate crop selection and local soil management.

## Conclusion

Under the conditions of this single-season field experiment in Torbat Heydarieh and Zaveh, post-saffron fields were associated with better subsequent crop performance than fallow fields for barley, cumin, chickpea, rapeseed, and sesame. The main response was an increase in seed yield across all five crops, with rapeseed showing the strongest yield response, while cumin provided the highest market-based economic performance. Biological yield followed a similar trend, suggesting that previous saffron cultivation may have had a positive residual influence on crop growth and biomass production. Among the studied crops, cumin had the highest market-based economic performance, followed by sesame and rapeseed. Therefore, these crops appeared to be the most promising options for cultivation after saffron within the studied regions. The comparison between locations showed that Zaveh generally provided more favorable conditions than Torbat Heydarieh, mainly because of lower salinity and better soil fertility status. In contrast, the relatively saline and alkaline conditions in Torbat Heydarieh highlight the need for careful soil fertility and salinity management. Overall, fields previously cultivated with saffron may provide a useful option for selected subsequent crops in the studied regions instead of remaining fallow, particularly when crop choice is adjusted to local soil conditions, crop adaptation, and economic value. Further multi-year and multi-location studies are needed to confirm the stability of these responses and to evaluate the longer-term effects of post-saffron cultivation on soil fertility, nutrient dynamics, microbial activity, and crop performance.

## Data Availability

All data supporting the findings of this study are available within the paper and its Supplementary Information.
